# Combined Population Dynamics and Entropy Modelling Supports Patient Stratification in Chronic Myeloid Leukemia

**DOI:** 10.1038/srep24057

**Published:** 2016-04-06

**Authors:** Marc Brehme, Steffen Koschmieder, Maryam Montazeri, Mhairi Copland, Vivian G. Oehler, Jerald P. Radich, Tim H. Brümmendorf, Andreas Schuppert

**Affiliations:** 1 Joint Research Center for Computational Biomedicine (JRC-COMBINE), RWTH Aachen University, 52062 Aachen, Germany; 2 Department of Hematology, Oncology, Hemostaseology, and Stem Cell Transplantation, Faculty of Medicine, RWTH Aachen University, 52074 Aachen, Germany; 3 Paul O’Gorman Leukaemia Research Centre, Institute of Cancer Sciences, College of Medical Veterinary and Life Sciences, University of Glasgow, Glasgow G12 0ZD, United Kingdom; 4 Fred Hutchinson Cancer Research Center, Seattle, WA 98109, USA; 5 Aachen Institute for Advanced Study in Computational Engineering Science (AICES), RWTH Aachen University, 52062 Aachen, Germany

## Abstract

Modelling the parameters of multistep carcinogenesis is key for a better understanding of cancer progression, biomarker identification and the design of individualized therapies. Using chronic myeloid leukemia (CML) as a paradigm for hierarchical disease evolution we show that combined population dynamic modelling and CML patient biopsy genomic analysis enables patient stratification at unprecedented resolution. Linking CD34^+^ similarity as a disease progression marker to patient-derived gene expression entropy separated established CML progression stages and uncovered additional heterogeneity within disease stages. Importantly, our patient data informed model enables quantitative approximation of individual patients’ disease history within chronic phase (CP) and significantly separates “early” from “late” CP. Our findings provide a novel rationale for personalized and genome-informed disease progression risk assessment that is independent and complementary to conventional measures of CML disease burden and prognosis.

Chronic myeloid leukemia (CML) is a clonal myeloproliferative disorder of a pluripotent hematopoietic stem cell (HSC) and the first human cancer that was found associated with a single consistent and causative molecular alteration, the oncogenic fusion tyrosine kinase BCR-ABL. Expression of BCR-ABL results from a reciprocal chromosomal translocation [t9;22 (q34;q11)] giving rise to the Philadelphia chromosome containing BCR-ABL1, the fusion of the breakpoint cluster region (BCR) gene and the Abelson tyrosine kinase (ABL1). BCR-ABL expression, with its constitutively active tyrosine kinase activity, is sufficient for the transformation of HSCs leading to CML^1^. Aberrant BCR-ABL signalling relays via a network of signalling pathways, reprogramming cellular signalling throughout chronic phase (CP), ultimately leading to myeloid hyperplasia and progression into accelerated phase (AP) and blast crisis (BC), if left untreated^2^,^3^. Potent treatment regimens have been established, yet hematopoietic stem cell transplantation remains the only cure^4^. For all other therapies, better response rates are achieved the earlier the disease is diagnosed and treated^5^. Despite progress made in the treatment of CML with tyrosine kinase inhibitors (TKI) since the advent of imatinib (Gleevec^®^), advanced disease still poses a problem, with lower treatment success and elevated relapse rates^6^. Prevention of disease progression remains the primary goal, and prediction of progression risk is of high clinical relevance. While disease stage classification has been refined via clinical, morphological, and genetic markers, the prediction of individual CP patients’ progressing risk is difficult to assess, with progression remaining a risk despite TKI treatment^7^,^8^. Prognostic scores such as Sokal, Euro (“Hasford”) or EUTOS are widely used in CML patient care. Despite being valid prognostication and prediction tools, these scores solely rely on established clinical parameters and still do not allow reliable inference of individual progression risk (Fig. 1a)^9^,^10^,^11^. Sokal and Euro scores were designed in the pre-imatinib era for improved comparison of clinical trial outcome. The Sokal score, however, has proven predictive for relapse-free survival after cessation of imatinib therapy, while the EUTOS was specifically developed to identify patients that do or do not benefit from imatinib^12^. Scores to predict failure of second generation TKIs, such as the “Hammersmith score”, have been developed and are in clinical use, despite limitations^13^.

Here, we focus on the need for novel biomarkers for individualized risk assessment. Dynamic CML progression modelling has largely been focussed on CP and the evolution of BCR-ABL ratios^14^,^15^. This allows only limited assessment of gene expression changes within the cell types implicated in progression and is not predictive of progression to AP or BC. Dynamic simulations focussed on imatinib therapy provided functional insights into the dependence of imatinib efficacy on the proliferative status of BCR-ABL-positive HSCs^16^. Model development for disease classification of first encounter patients requires modelling in the untreated state. While this approach suffers from a lack of longitudinal data, clinical validation is mostly impossible, since patients are routinely subjected to TKI treatment upon diagnosis. Therefore, despite statistical complications, utilization of retrospective datasets represents a supplementary approach. Gene expression profiling has enabled unsupervised analyses of progression stages^17^.Although recent results indicate a reliable patient characterization using supervised gene expression biomarker identification, they failed to show sufficient reliability for clinical use^18^. Still, genome-wide differential expression based biomarkers display a remarkable stability across heterogeneous studies of clinical samples^19^,^20^. Gene expression measurements will enter future CML diagnostic routine, as has been shown for breast cancer, where gene expression assays provide clinically useful prognostic information^21^.

To address clinical need of CML patient status and risk assessment with respect to disease evolutionary time, we evaluated the utilization of retrospective, non-longitudinal data from untreated patients to derive patient-specific genomic progression scores. We propose a novel approach to overcome statistical complications arising from supervised analysis of multivariate “omics” datasets and to address limitations of existing models. Figure 1 outlines the overall motivation and analytical strategy of our approach. In contrast to former strategies, our approach relies on the integration of an established CML population dynamic model with two patient-derived genomic biomarkers: firstly, a biopsy genome-wide expression-derived CD34^+^ similarity score as indicator of disease history and progression risk; and secondly, gene expression entropy as indicator of instability during critical states (Fig. 1b). The approach recapitulates patient classification according to established clinical stages and parameters. Also, our strategy reveals additional heterogeneities amongst CP patients and suggests a separation of “early” versus “late” CP, which lends itself to progression risk assessment.

In order to infer patient status from genomic profiles and CML population modelling, we combined scores obtained from an established model with the two corresponding patient-derived genomic scores^17^,^15^ (Fig. 1c). We calculated patients’ CD34^+^ similarity status, where expression of the differentiation marker CD34 is high in stem cells but lost during differentiation. The corresponding model parameter CD34 ratio reflects these cell population changes during CML hematopoiesis, where, over time, CD34^+^ blasts outnumber differentiated and committed CD34^−^ cells. CD34 ratio is correlated with patient CD34^+^ similarity scores, connecting CP patient disease status with disease progression time in the model. Additionally, we considered entropy as a quantitative measure of disorder and gene expression de-regulation. The non-monotonic evolution features of modelled entropy served as an alignment marker to map patient gene expression and disease time. Here, we relied on the published time span of six years between the occurrence of the first CML HSC and clinical presentation of the disease as an established generalization in order to introduce our mapping concept^15^. Our integrated approach extends existing modelling concepts and addresses clinical need by enabling disease status approximation with respect to disease evolutionary time. Despite existing limitations, this relation of patient genomic status to disease time represents a methodological innovation in addition to conventional clinical scores that is not restricted to CML, but holds potential for predictive modelling in a variety of other malignant diseases with seemingly homogenous extended chronic phases, especially those with a risk of progression into lethal stages that are often unresponsive to treatment.

## Results

### Separation of population-based and mechanistic effects using stem and progenitor cell data from primary CML patients

Established computational models describing CML progression dynamics simulate disease evolution in terms of hematopoietic population growth over time^15^,^16^. To directly assess CML disease stages with respect to disease evolutionary time, we aimed at reconciling patient gene expression with an established model of CML evolution by Dingli and co-workers (Supplementary Resources and Methods)^15^. First, to investigate the degree to which CML progression is driven by hematopoietic cell population dynamics as opposed to differences in the genetic underpinnings of respective disease stages, we analysed GEO dataset GSE47927, comprising data obtained from CD34^+^ enriched, flow sorted CML stem and progenitor cell populations from 12 CML patients, of which six CP, four AP, and two BC, versus matching populations from three healthy volunteers (Supplementary Resources and Methods)^22^. We visualized gene expression patterns by principal component analysis (PCA) considering the information-rich subset of the human genome of ~6,000 genes with high predictive accuracy regarding clinical phenotypes and stable biomarkers during meta-analysis of clinical data sets^20^ (Methods). The first three PCs revealed differential expression, clearly separating HSCs, common myeloid progenitors (CMPs), granulocyte-macrophage progenitors (GMPs), and megakaryocyte-erythroid progenitors (MEPs) (Fig. 2, Supplementary Fig. S1). CML HSCs display an increase along PC2 compared to normal HSCs. This clear separation between CML and normal HSCs along PC2 is less pronounced for normal versus CML CMPs, and is not apparent in GMPs or MEPs, indicating that differential gene expression, evident by separation along PC2, decreases with hematopoietic differentiation. Up-regulated genes in PC2 include CSF1, CSF2RB, CD38, CD36, and FCGR2A, and down-regulated genes include CD34 and CD79B, suggesting that CML HSCs are phenotypically more differentiated towards the myeloid lineage than normal counterparts. PC1 and PC2 parameters are therefore able to detect BCR-ABL - induced changes in HSCs. Comparing these trends to a projection of the gene expression from clinical samples of patients in different CML disease stages (dataset GSE 4170)^17^ suggests that genome-wide differential expression in CML is driven by population- based differentiation processes rather than mechanistic effects linked to disease progression (Supplementary Fig. S2).

### Discerning CML disease stages by CD34 status of patient gene expression

We hypothesized that gene expression associated with differentiation could serve as an indicator of hematopoietic cell population changes associated with CML progression. During healthy hematopoiesis, the surface marker CD34 distinguishes immature HSCs and progenitor populations (CD34^+^) from committed precursors and differentiated cells (CD34^−^). An expansion of CD34^+^ immature blasts is a hallmark of CML progression^23^,^24^. The increase of immature CD34^+^ over differentiated CD34^−^ cell populations entails an increasing CD34 ratio with disease progression. We inferred the CD34 ratio according to equation (1), as a corresponding model parameter from an established model of multi-compartmental hematopoiesis^15^. Since CD34 ratio increases over time in accordance with this model, we hypothesized that we can use it to link patient status, examined at hand of patient-specific CD34 expression scores, in order to map patients to CML disease time in the model.

We assessed CD34 expression in mixed-population clinical samples of CP (42), AP (9), AP_cyto_ (8), and BC (28) patients (dataset GSE4170)^17^ to obtain CD34^+^ similarity scores as a corresponding patient parameter and indicator of disease status (Supplementary Resources and Methods). CD34^+^ similarity scores represent hematopoietic differentiation status over time in healthy individuals and CML patients. Combining patient-derived genomic information with the conventional parameter blast count, we show that CD34^+^ similarity adds an additional dimension of separation of established CML stages. While separating CP from advanced stages (AP, BC), blast count alone does not distinguish CP cases amongst each other, or from AP_cyto_ cases, which are characterized by additional cytogenetic changes only. CD34^+^ similarity, however, separates CP, AP_cyto_, AP and BC cases, indicating increasing similarity of patient gene expression to CD34^+^ naïve cells with disease progression, and distributes CP patients over more than one order of magnitude, suggesting differences during CP evolution that are not resolved by blast count alone (Fig. 3a). Importantly, patient CD34^+^ similarity scores correlate significantly with model-derived CD34 ratios (R^2^ = 0.934, p = 3e-25) (Supplementary Fig. S3). This correlation represents a monotonic map between disease progression time in patients and in the model, which we assume to be linear in CP. Since the parameters are not known *a priori*, a second progression marker is required, whose dynamics are not correlated and as such independent of the first marker, CD34 status.

### Entropy of gene expression as indicator of disease progression

We assessed Shannon entropy according to equation (2) as a quantitative measure of the disorder and lack of co-regulation of gene expression. Simulated entropy of randomized gene expression displays a non-monotonic dynamic trend with a singular minimum. Equally, simulated entropy of cell population mixing throughout hematopoietic evolution (mixing entropy) shows a singular maximum aligning with the minimum of simulated entropy. We conclude that lowest gene expression entropy coincides with highest heterogeneity of cell populations (cell population mixing effect), whereas entropy is higher for homogeneous cell types (Supplementary Fig. S4, Methods). We therefore consider low gene expression entropy as an indicator of population heterogeneity, instability, and disease progression. Both entropies are related to the time scale of disease evolution in the model. Comparing patient-derived and simulated entropies, we find significant correlation (p = 0.0195). Challenging the significance of this correlation through random sub-sampling reveals a frequency distribution with p values < 0.05 for a majority of 80.2% of iterations (Supplementary Fig. S5). Next, we assessed patient gene expression (GSE4170) entropy in combination with CD34^+^ similarity in order to combine resolution provided by both markers. Compared to the separation of CML disease stages by CD34^+^ similarity and blast counts, entropy reveals further differences, especially amongst CP cases, with high entropy variability over the same CD34^+^ similarity range (Fig. 3b). We observe a V-shape in patient distribution as a result of two trends: with increasing CD34^+^ similarity, early CP cases show decreasing entropy, then entropy starts to incline again. Second, continued entropy increase with increasing CD34^+^ similarities corresponds to progression into AP and BC. We find that AP and AP_cyto_ entropies are overall elevated and that BC entropy is increased with respect to CP, indicating loss of genome-wide gene expression regulation along progression. Advanced CML is located to the right side of the entropy minimum, AP patients starting to emerge first, followed by BC patients. We conclude that AP cannot be described by mere mixing of CP and BC, but that it rather represents an intermediate stage with characteristic expression features^17^. Four putative CP outliers at entropy >2 are in CD34^+^ neighbourhood of some AP_cyto_ cases. Entropy >2 is characteristic for eight out of nine AP_cyto_ cases, whereas the majority of CP cases have entropies <2. We hypothesized that these CP cases represent early AP_cyto_ patients not classified as such by conventional methods. Comparing their gene expression against CP cases in the same CD34^+^ similarity range albeit with lower entropy (< 2), we found significant differential expression (p-value < 0.01, FDR-adjusted p-value), whereas comparison to AP_cyto_ samples in the same range did not reveal significant differences (p > 0.01), supporting their similarity by genomic features. Since these p values are based on the present patient cohort only, they should be considered as indicators. Positioning along the entropy axis identified a sub-population of CP patients as putative “early” AP_cyto_ cases with signs of early onset of disease progression.

### Assessing CP CML status and progression risk

**Figure 1:**
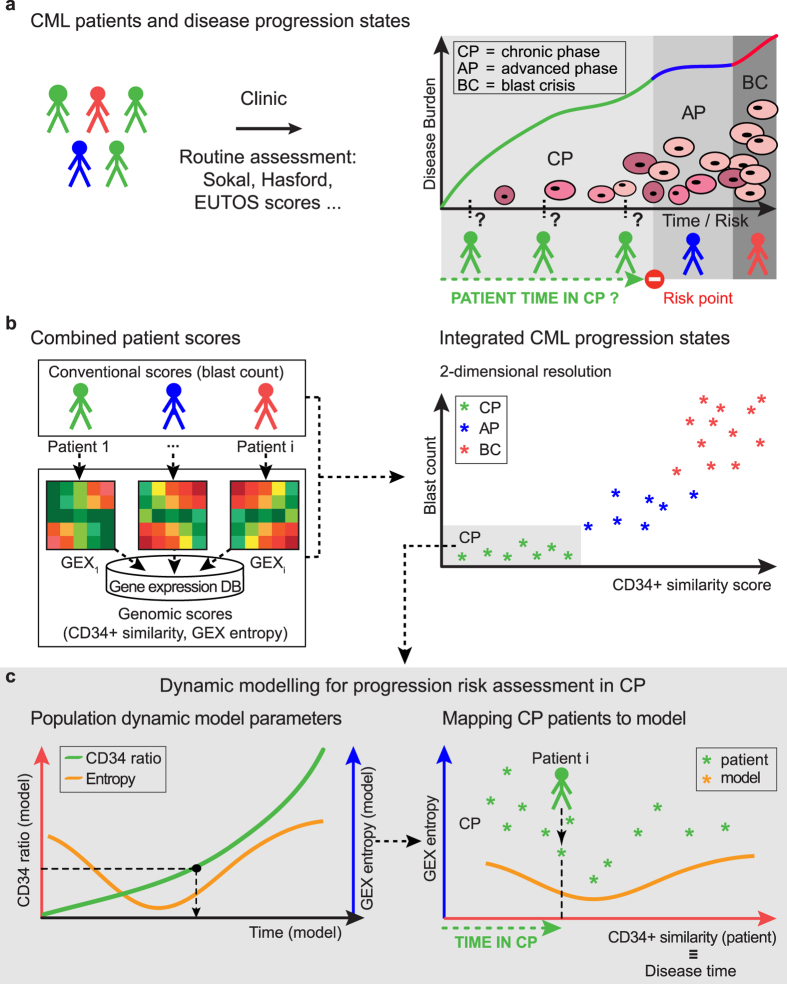
Analysis Workflow. Workflow outlining rationale and analytical pipeline. (**a)** Established clinical parameters classify CML patients in disease progression states regardless of genomic profile. (**b)** Combination of conventional scores, e.g. blast count, with patient gene expression (GEX) - derived genomic scores “CD34^+^ similarity” and “entropy” enables patient characterisation according to complementary scores at increased resolution. CD34^+^ similarity indicates disease history and progression risk, entropy is a quantitative measure of disorder or lack of GEX co-regulation. (**c)** Focus on CP patient progression risk assessment according to the CML population dynamic model by Dingli *et al.*^15^. The model parameter CD34 ratio reflects cell population dynamics and is correlated with patient CD34^+^ similarity scores. Non-monotonic evolution of the model parameter entropy serves as alignment marker to map patient gene expression and disease time within CP. CP = chronic phase, AP = advanced phase, BC = blast crisis of CML.

**Figure 2:**
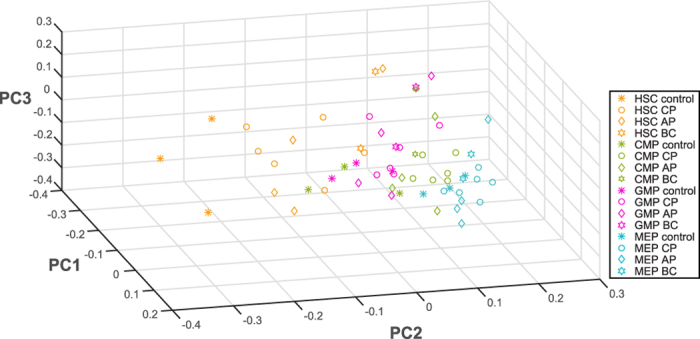
Separation of population-based and mechanistic effects using stem and progenitor cell data from primary CML patients. Genome-wide gene expression of 4 stem- and progenitor cell subpopulations (colours) across all phases of CML progression (shapes) in 12 patients and three healthy controls, represented by PCA-derived components 1, 2, and 3. PC: Principle Component, CP: chronic phase, AP: accelerated phase (by blast count criteria), AP_cyto_: accelerated phase (by occurrence of additional clonal cytogenetic changes without increase in blast count), BC: blast crisis, HSC: hematopoietic stem cell, MEP: megakaryocyte-erythroid progenitor, GMP: granulocyte-macrophage progenitor, CMP: common myeloid progenitor. Data from GEO dataset GSE47927, by Copland M and Irvine DA^22^.

**Figure 3:**
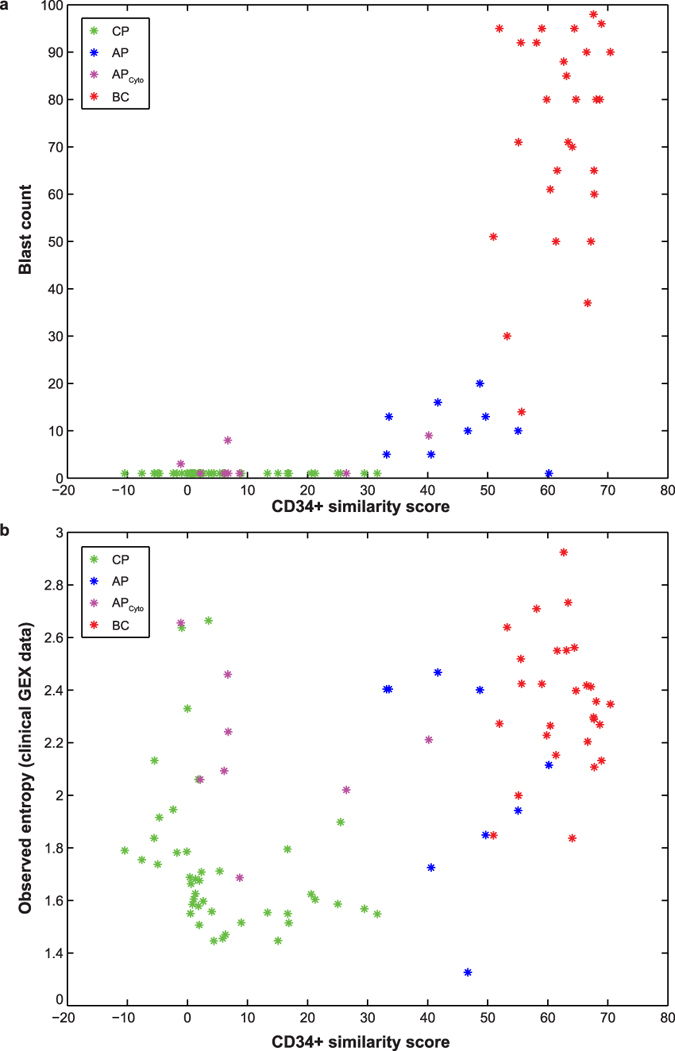
Integrated tracking of CML disease progression stages from mixed-population clinical samples. **(a)** Data points represent clinical samples of 42 CP (green), 9 AP (blue), 8 AP_cyto_ (purple), and 28 BC (red) patients^17^. Each asterisk represents one patient. Differences between patients and corresponding disease stages are resolved by CD34^+^ similarity score in combination with blast count. (**b)** Data points as in a. Entropy of patient gene expression resolves differences in disease stages in combination with CD34^+^ similarity score.

**Figure 4:**
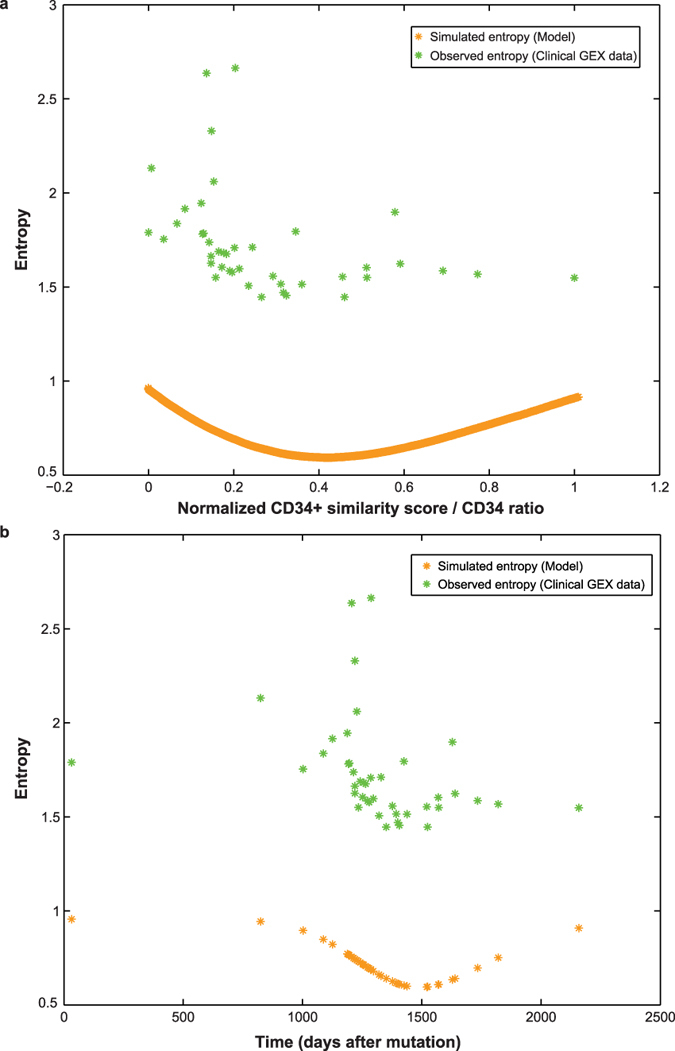
Assessment of CP patient disease status and progression risk. (**a)** Data points represent clinical samples of 42 CP patients (green asterisks) (GSE4170, Radich *et al.* 2006a). Normalized CD34^+^ similarity score combined with entropy of patient gene expression and simulated entropy maps CP evolutionary time intervals between model and patients. (**b)** 42 CP patients (green asterisks) as in a. plotted by observed entropy from clinical gene expression data and simulated entropy from the model converted to time (days after initial HSC mutation). Timespan covers ~6 years in accordance with the model.

To address the challenge of disease status identification and progression risk prediction during CP CML, we turned to the mapping of CP patient status to disease progression time^15^. Since gene expression entropy is directly related to CML evolutionary time through the model, we used the non-monotonic evolution features of entropy as an alignment marker to map patient gene expression and disease time (Fig. 1c). Entropy introduces a significant V-shape (p = 0.014788, linear regression) within CP samples when distributed along CD34^+^ similarity (Fig. 4a, Methods). To match model and patient data, we normalized CD34^+^ similarity scores and the CD34 ratio to the interval [0, 1] to highlight similarity of entropy evolution between clinical data and model simulation (Fig. 4a, Supplementary Fig. S6). Since CD34^+^ similarity corresponds to CD34 ratio, and therefore to disease time according to the model, we mapped normalized ‘observed entropy – simulated entropy’ pairs to a time interval of ~6 years (2,200 days) as a general approximation to the average disease behaviour across a heterogeneous patient population, and in accordance with the modelled duration from the initiating mutation until diagnosis (equation (3)). In order to align the minima observed in patient gene expression entropy and in simulated entropy, we pinned corresponding data points by finding minimum entropy distance between each pair (Fig. 4b, Supplementary Fig. S6, Methods). Herein, we present an integrated modelling strategy for the translation of clinical stage to true time scale of disease evolution, leveraging the combined power of CD34 status and entropy parameters.

**Figure 5:**
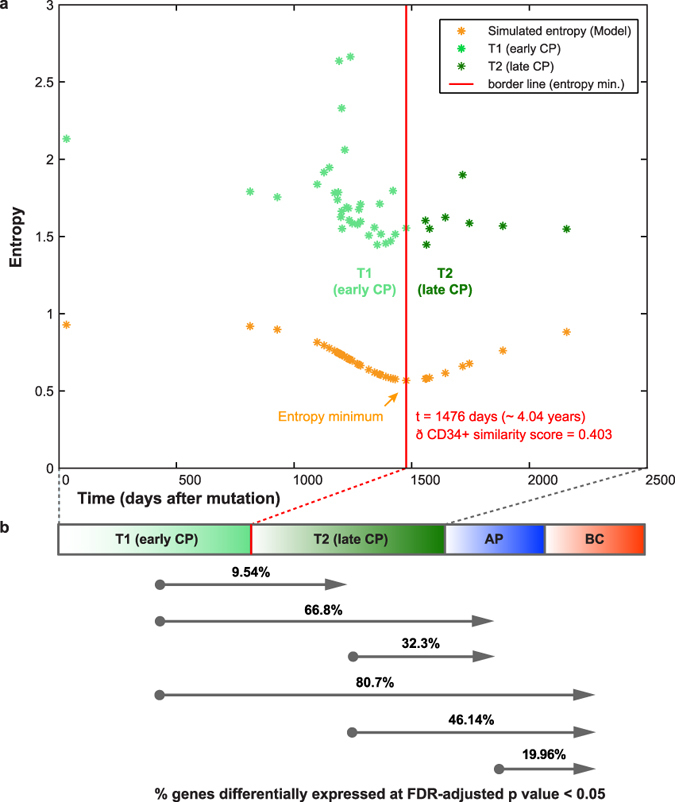
Entropy-based separation of early vs. late chronic phase (CP) patients. **(a)** Integrating CD34^+^ similarity score with entropy of patient gene expression and simulated entropy resolves differences in patient disease evolutionary time. 42 CP patients (asterisks) plotted by observed entropy from clinical gene expression data aligned by model-derived simulated entropy converted to disease time span (days). Time span covers ~6 years in accordance with the model. The red borderline separates “early” (T1) from “late” (T2) CP patients (light and dark green asterisks, respectively) at the simulated entropy minimum singularity at *t* = 1476 days (~4.04 years), and CD34^+^ similarity score = 0.403. **b.** T1 and T2 CP patient groups are significantly different in terms of gene expression (T1 vs. T2, FDR-adjusted p < 0.05). Progression towards advanced stage is highlighted by increasing differential gene expression compared to AP and BC, where the fraction of differentially expressed genes of T1-CP vs. AP (66.8%) > T2-CP vs. AP (32.3%), and T1-CP vs. BC (80.7%) > T2-CP vs. BC (46.14%).

We hypothesized that differences between CP patients resolved by CD34^+^ similarity and entropy could indicate an early disease progression trajectory. Following normalization and patient mapping to model-derived disease evolutionary time, we considered the simulated entropy minimum singularity as an indicator for a critical disease state transition during CP and separated “early” (T1) from “late” (T2) CP patients at the entropy minimum borderline at *t* = 1476 days, corresponding to ~4.04 years and a CD34^+^ similarity score ~0.403 (un-normalized score = 13.34) (Fig. 5a). While patient CD34^+^ status is assessed from genomic data, the corresponding 4-year time interval is restricted to the model parameters. This time point is directly dependent on calculation of the CD34 ratio at the exemplary cut-off at compartment 23, from which committed precursor cell types dominate over undifferentiated progenitors according to the multi-compartment model^25^. Testing the impact of parameter variations on this boundary, we calculated CD34 ratios across all possible cut-offs for CD34^+^ expression and observed a robust T1–T2 boundary around 4 years (Supplementary Fig. S7, Supplementary Resources and Methods).

### Genomic differences confirm early disease progression during CP CML

We next assessed differential expression between the thirty-four T1 and eight T2 CP patients and identified 411 differentially expressed genes (FDR-adjusted p-value < 0.05), indicating that T1 and T2 CP differ by differential expression of almost 10% of genes, compared to ~20% when comparing AP vs. BC (Fig. 5b, Supplementary Fig. S8). The ratio of differential genes between T1-CP and AP (66.8%) is >2-fold increased compared to T2-CP and AP (32.3%), clearly distinguishing T1-CP and T2-CP with respect to progression towards AP, and highlighting the significance of the two groups in terms of disease progression within CP. This difference is upheld when compared against BC patients. T1-CP and BC differ by differential expression of 80.7% of genes, compared to 46.14% between T2-CP and BC. These unexpected, profound effects within CP suggest that T2-CP may represent a higher risk state with respect to progression. We confirmed robustness of these substantial fractions with respect to changes in cohort size by random sub-sampling analysis (Supplementary Figs. S9 and S10, Supplementary Resources and Methods).

Assessing differences in biological processes by Gene Ontology (GO) enrichment amongst the 411 genes differentially expressed between T1- and T2-CP, we found 91 biological processes significantly enriched for down-regulated genes, and 228 terms for up-regulated genes. While GO terms enriched amongst up-regulated and down-regulated genes overlap with 65 common terms, 26 processes were exclusive to down-regulated genes, and 163 were exclusive to up-regulated genes (Supplementary Tables S1 and S2). Several processes frequently observed amongst both up- and down-regulated genes are represented at approximately equal proportions, such as terms related to protein translation, modification or trafficking (15% in up- vs. 12% in down-regulated genes), and metabolism, biosynthesis or catabolic processes (11% vs. 10%, respectively). Strikingly, 31% of GO biological processes exclusively enriched amongst down-regulated genes pertain to innate immune response regulation, while no GO process of this category is enriched in the jointly differentially regulated set or amongst up-regulated genes. Also, 15% of processes exclusively enriched amongst down-regulated genes are related to cellular differentiation and development. On the other hand, 13% of GO terms enriched exclusively amongst up-regulated genes are related to signal transduction involved in DNA damage, integrity checkpoints or general cell cycle control. These over-representations are in concordance with cancerogenic processes, including innate immune responses to changes in tissue homeostasis, impaired cellular differentiation, and DNA damage response signalling upon genomic instability, underlining the significance of the progression trend between T1- and T2-CP^26^,^27^,^28^.

## Discussion

CML represents a targeted cancer therapy paradigm and served as foundation for the first computational models providing insights into *in vivo* kinetic behaviour of a human cancer. In CML, BCR-ABL alone induces a monoclonal malignancy with a strong phenotype. Tumour cells remain dependent on BCR-ABL and can be forced into apoptosis by targeted inhibition with TKIs such as imatinib, while CML HSCs appear insensitive^29^,^30^. Pioneering computational modelling of CML kinetics during imatinib therapy revealed a biphasic exponential decline of leukemic cells, where the first slope corresponds to differentiated cells and the second slope to leukemic progenitors, suggesting that leukemic HSCs are not depleted^14^,^31^. This model is challenged by evidence based on outcome of CML patients undergoing imatinib mono-therapy, according to which long-term clinical response data are consistent with models considering a primary imatinib effect on proliferating BCR-ABL1-positive HSCs, when committed into the cell cycle^16^,^32^. These studies highlight the importance of competition between normal and leukemic cells for CML therapy^33^. Recent evidence corroborates that CML cells outnumber normal cells, promoting proliferation, altering differentiation, and inhibiting self-renewal capacity of non-transformed progenitors via interleukin 6^ ^34. Even though competition effects ought to aggravate the differentiation block, exacerbating the CD34 ratio shift, our model may underestimate the effect of CML cells, as the data we considered lacks a separation of healthy vs. CML cells. Our approach suffers from the limitation that competition effects cannot be considered. Also, we cannot address heterogeneity introduced through diverging mutational patient profiles or varying proliferation rates. While intrinsic model time can at most address an average patient population, our approach uses individual progression scores to match patients to the model, leaving classification results unaffected by patient variation in proliferation rate. Leukemias are considered less complex regarding the number of mutations compared to the majority of solid tumours^35^. Therefore, despite these limitations and controversies, CML represents a suitable model disease, especially useful for the development of predictive models aimed at clinical translation.

To date, CML models have either focused on the simulation of generic disease progression within CP under therapy, or on characterizing individual patients’ progression. Despite advancements in CML treatment, fundamental challenges in disease management remain. Following a homogenous and extended CP, a minority of patients evolve more aggressive myeloid neoplasia with lower drug response and higher relapse rates^5^,^36^. Even during TKI therapy, patients are at risk of disease progression, predominantly during the first two years^37^, suggesting that affected patients may have been diagnosed in “late” CP. Accelerated telomere shortening has been proposed as a prognostic CML marker^38^,^26^. While this process might be attributed to an inflammatory environment^39^, as shown for other malignant diseases^40^, we did not observe increasing inflammatory gene expression during CP progression. Gene expression data from unsorted, bone marrow - derived CML cells challenged the three-phase progression concept, suggesting that AP may not represent a discrete stage^17^, whereas our results support the concept that AP represents a transition state. Considering CML dynamics we conclude that precise, personalized staging and risk assessment based on genomic and molecular parameters should inform clinical strategy. The need for precise staging within CP remains a core issue of clinical relevance. How long has the disease persisted prior to diagnosis? What is the patient’s time from transition into advanced disease?

**Figure 6:**
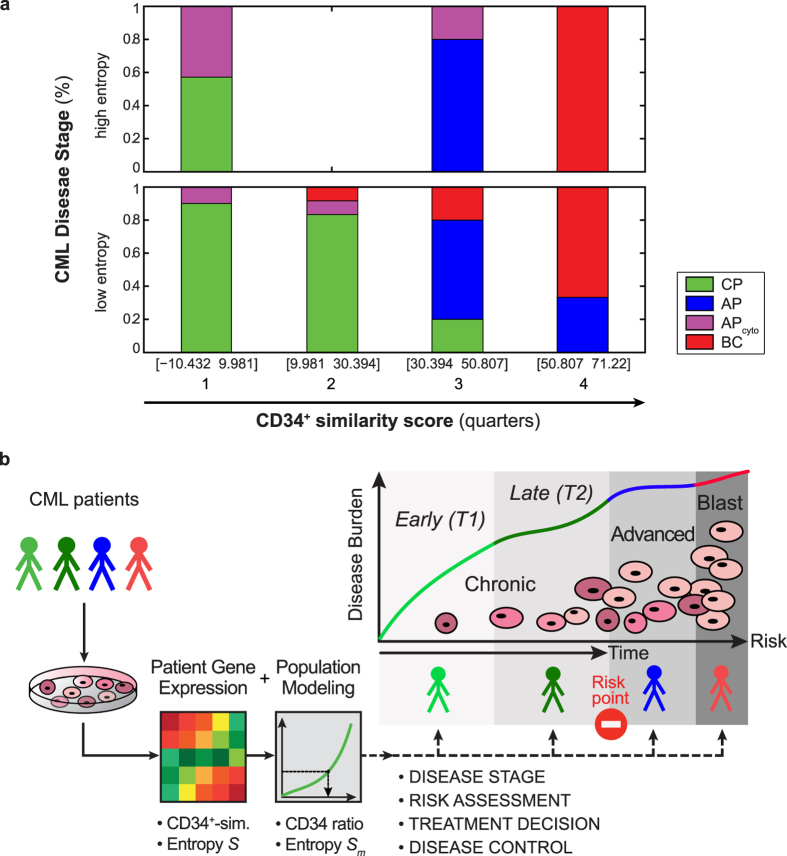
Integrated concept for CML patient stratification and risk assessment during chronic phase (CP). (**a)** Disease stage fractions (%) observed when patients are subdivided into CD34^+^ similarity quarters and low vs. high entropy, yielding eight sub-spaces. Graph represents disease stage fraction of each sub-space compared to all patients (42 CP (green), 9 AP (blue), 8 AP_cyto_ (purple), and 28 BC (red) patients^17^, as in Fig. 3). **(b)** CML patient biopsy gene expression profiling enables differentiation of patients by CD34^+^ similarity and gene expression entropy. A population dynamic model for quantification of the evolution of hematopoietic cell types across CML serves to identify dynamics that are characteristic for disease status. Upon entropy-mediated alignment to time scale of disease evolution during CP, patient status can be matched to disease evolutionary time for risk assessment and personalized therapeutic intervention.

To this end we present a novel approach for characterization of patient status along disease evolutionary time. Firstly, our focus on CP is justified by the availability of population dynamic models, higher therapeutic success rates, and the need to prevent progression. Within clinically homogenous stages, identification of appropriate parameters to assess disease evolution is not straightforward. Secondly, our integrated approach addresses this shortcoming and aims to improve predictive modelling. We show that CD34^+^ similarity scores achieve patient separation along CML progression, revealing differences within CP that are not resolved by blast count alone. We observed linear correlation between patient-derived CD34^+^ similarity and CD34 ratio, the corresponding model parameter. Motivated by the concept of phase transitions of complex systems in statistical thermodynamics^41^, we assessed Shannon entropy of gene expression to reveal critical states that can connect patient status and model disease over time. We observed characteristic entropy singularities, where low gene expression entropy relates to high cell population heterogeneity, instability, and disease progression, indicating that quantitative concepts from phase transition theory can be utilized as complementary factors in biomedicine for the development of quantitative disease models. We introduced entropy as an alignment marker to retrospectively match CP CML patient status to model disease progression time (“pinning”)^17^. Both markers, CD34 status and entropy, are strictly complementary, together achieving a new level of resolution for genome-informed patient stratification. Quantifying patient fractions within disease stages throughout both dimensions enables estimation of disease stage likelihood (Fig. 6a). This quantification recapitulates the established progression trajectory from CP via AP to BC, and highlights differences not revealed at the cellular level alone. While ~90% of patients are in CP at low CD34^+^ similarity, this fraction decreases to 60% with concomitant rise of ~40% in AP_cyto_ at the same CD34^+^ similarity and high entropy. These disease stage fractions are not observed by chance. Analogous and randomly staged patient cohorts would suggest about 40–50% CP, 10% AP, and 30–40% BC are in the first CD34^+^ similarity quarter at low entropy. In reality, however, 90% of patients are in CP, and no patients are in AP or BC in the clinical cohort. (Supplementary Fig. S11). The second quarter marks a low entropy phase, indicative of a transition state. In the third, CP is found less frequently than expected randomly, and more patients than expected by chance are annotated as AP. High entropy and high CD34^+^ similarity scores characterize 100% patients as BC, whereas only 30–40% are expected by chance. This quantification lends itself as supporting rationale for genomics-based risk assessment in CML.

In summary, our approach i) relates patient status to disease time scale inferred from a population dynamic model, ii) provides a new way to approximate progression risk, and iii) reveals new insights into pathophysiological CML dynamics. Entropy dynamics suggested a clear subdivision of CP into early (T1) and late (T2) stages with significant gene expression differences compared to advanced disease. Functionally, GO enrichment revealed broad genomic differential expression across hundreds of biological processes, pointing towards systematic changes during CML progression in consequence of BCR-ABL signalling, rather than deregulation of a single or defined set of signalling pathways. Enrichment of innate immune response processes amongst down-regulated genes points towards a compromised immune response and impaired tumour surveillance. While innate immunity lacks the adaptive response’s antigen recognition, natural killer cells can target and eliminate cancer cells; a possible target mechanism for cancer immunotherapy^42^. Down-regulation of differentiation and development genes indicates impaired hematopoietic differentiation implicated in proliferative advantages of transformed cells. Enrichment in up-regulation of DNA damage, integrity checkpoint and cell cycle processes is indicative of responses to enhanced DNA damage and genomic instability, both implicated in disease progression and drug resistance^36^. Overall, reduced immune surveillance, impaired differentiation, and exacerbated genomic instability are hallmarks of cancer, reflected here by differential expression between two CP sub-groups.

We suggest that a normalized CD34^+^ similarity of ~0.4, or an interval of ~4 years from the initiating mutation, represents a risk point for disease progression. While patient CD34^+^ similarity status can be assessed directly from patient genomic data, the corresponding 4-year time interval is an approximation restricted to the model. As evidence and established models suggest ~3.5 to 6 years for the disease to become clinically apparent, our data lead us to conclude that after ~4 years CML enters an advanced stage of CP. Patients staged beyond this point could be subject to increased progression risk (Fig. 6b). Taken together, our model enables quantitative interpretation of a combination of conventional and genomic markers in terms of disease evolutionary time. Our observations establish direct links between patient gene expression and the underlying hematopoietic population heterogeneity, positioning our approach as a novel exploratory tool with potential for wide applicability in clinical research. This potential extends not only to the complete spectrum of CML stages, but also to localized and metastatic solid tumours, other leukemias, or serious, malignant diseases with chronic stages at risk of fatal progression, such as certain myeloproliferative neoplasms, where no mathematical models or predictive biomarkers exist to date.

## Methods

### Data Source and Preparation

To study hematopoietic cell population-based effects during CML disease progression we analysed GEO dataset GSE47927, comprising gene expression data obtained from four CD34^+^ enriched and flow sorted CML stem and progenitor cell subpopulations (HSC: hematopoietic stem cell, MEP: megakaryocyte-erythroid progenitor, GMP: granulocyte-macrophage progenitor, CMP: common myeloid progenitor) across all phases of CML progression (CP: chronic phase, AP: accelerated phase (blast count), AP_cyto:_ accelerated phase (additional clonal cytogenetic changes without increased blast count), BC: blast crisis) from 12 patients, of which six CP, four AP, and two BC, versus matching populations from three healthy volunteers^22^. For the identification of features associated with genetic underpinnings and mechanistic effects of disease progression, we used GEO dataset GSE4170 involving genome-wide gene expression data from 42 CML patients in CP, 9 in AP, 8 in AP_cyto_, and 28 in BC^17^. CP patients with missing blast count information were assigned blast count = 1. Four BC patients with missing blast count information were not considered in the analysis. For principal component analyses using the gene expression datasets above, we started with a subset of the human genome of 6,384 genes that are characterized as information-rich by a low information ratio (IR), corresponding to predictive accuracy with respect to clinical phenotypes and stable biomarkers^20^. We then considered the subset of low-IR genes represented as complete cases in the respective gene expression datasets, which are 6,078 genes for dataset GSE47927, and 4,309 genes for GSE4170. All computations were performed in MATLAB (The Mathworks Inc, Version 14, Statistics Toolbox). For further details on the associated GEO datasets please refer to the Supplementary Resources and Methods.

### Calculating CD34^+^ similarity scores from patient gene expression data

We calculated CD34^+^ similarity scores from patient data^17^ using the PhysioSpace method as described^43^. Briefly, we assembled a CD34^+^ reference by calculating mean gene expression over all CD34^+^ samples obtained from published dataset GSE4170. For this reference sample we then ranked the genes according to their expression level and extracted the top 5% most up- and down-regulated genes as representative CD34^+^ status associated genes. We then calculated the CD34^+^ similarity score of each sample as signed log10-p-value obtained from Wilcoxon rank sum testing between the top 5% up- and down-regulated CD34^+^ status associated genes.

### Modelling CD34 ratio

The population dynamic model by Dingli and co-workers considers hematopoiesis as a hierarchically structured process comprising 32 compartments^25^,^15^. During healthy hematopoiesis, stem cells (HSCs), progenitor and some precursor cells express CD34 surface marker (CD34^+^), while some precursors and terminally differentiated cells lose CD34 expression, and mature cells representing the majority of the hematopoietic cell mass are CD34^−^. During CML, however, a differentiation block causes population shifts to a highly abundant pool of CD34^+^ leukemic blasts, shifting the ratio of CD34^+^/CD34^−^ (CD34 ratio). This increase in CD34 ratio can be inferred according to the model by Dingli *et al.* as follows:


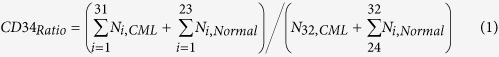


In order to integrate CD34^+^ similarity scores derived from patient gene expression data^17^ with the CD34 ratio obtained from the mechanistic model into a unique variable, we assume linear correlation through a monotonically increasing function.

### Assessing patient biopsy genome-wide expression entropy

In order to calculate the Shannon-entropy for each patient sample k according to:


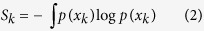


we used original data from^17^ to calculate the distribution density p(x_k_) in sample k for the information-rich subset of ~6,000 genes of the human genome that are characterized by a low information ratio (IR)^20^. We assessed the V-shape resulting from entropy differences as a dependent variable with respect to CD34^+^ similarity as an independent variable in CP CML patients using linear regression modelling using the ‘fitlm’ function with quadratic term (MATLAB Linear Regression Package).

### Model-based simulation of gene expression entropy

To calculate the simulated gene expression entropy based on the population dynamic model^15^, we generated a randomly simulated gene expression matrix SGEM such that the number of rows is equal to 64, as we consider each 32 compartments of healthy and CML cells. The number of columns is identical to the number of genes (6,384 low IR genes) considered from patient gene expression data. Rows represent expression of the ~6,000 genes in each cell population (compartment). In order to simulate the gene expression dynamics of a mixed population of cell types, we weighted each gene in the SGEM according to the cell population dynamics during CML evolution in CP, which were inferred from the population dynamic model. We thereby obtained expression changes of each gene in a mixture of healthy and CML cells during CP evolution. We calculated the simulated gene expression entropy as Shannon-entropy of this final product (s_t_).

### Mapping clinical data to time scale of disease evolution for CP patient disease progression risk assessment and stratification

In order to match patient samples to time of disease progression we aligned entropy profiles derived from model simulations and patient gene expression, considering CD34 ratio to be a surrogate for time, as follows:


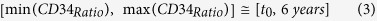


Second, we independently normalized patient-derived CD34^+^ similarity score and simulated CD34 ratio to the same interval [0 1]. Then, considering linear correlation between CD34 ratio and CD34^+^ similarity score (Supplementary Fig. S3), we observed similarities in evolution of entropy derived from patient gene expression compared to simulated entropy (Fig. 4a). This normalization enabled mapping of CP CML patient samples within the time scale of disease evolution in a quantitative way.

In order to perform this mapping we first identified the corresponding CD34 ratio for each individual patient by calculating the distance between each patient’s normalized CD34^+^ similarity score and all possible normalized CD34 ratios. We then identified the nearest simulated entropy point in order to obtain pairs of minimum distance between observed (patients) and simulated entropy (model). Since each CD34 ratio is equivalent to a unique time point according to the model, we obtain a corresponding time point for each patient. Thereby we aligned both minima of simulated and patient – derived entropies (“pinning”), such that each CP patient is at minimum distance to its corresponding simulated data point in both independent scores (Fig. 4b).

### Analysis of differential expression along disease progression stages

To assess differential gene expression patterns between disease stages we considered the set of 4,309 genes represented as complete cases in dataset GSE4170 that were also amongst the 6,384 genes with low information ratio (IR) as described^20^. A cut-off of p < 0.05 (FDR-adjusted p-value) was used to identify differentially expressed genes between two groups. Significance of differential expression was assessed by 2-sided t-test (MATLAB statistical toolbox).

### Gene Ontology enrichment analysis

Gene Ontology (GO) enrichment analysis was performed on each set of significantly differentially up- or down-regulated genes between early (T1) and late (T2) CP. Since analysis of differential gene expression was performed by 2-sided t-test, the differences between the mean of T1-CP and T2-CP distributions classified each gene as up- or down-regulated. GO enrichment analysis was carried out according to the PANTHER Overrepresentation Test (release 20150430) against the reference set of 4,309 genes from dataset GSE4170 that were represented amongst the 6,384 genes with low information ratio (IR) (see above)^20^, considering the GO Ontology database released 2015-08-06, including Bonferroni correction for multiple testing. Biological processes with p < 0.05 were considered and ordered by fold enrichment.

### Disease stage quantification and patient cohort randomization control

In order to quantify how CML disease stages (CP, AP_cyto_, AP, and BC) partition amongst the patient cohort, CD34^+^ similarity and gene expression entropy ranges were evenly divided into quarters and halves (low vs. high entropy), respectively, dividing the CD34^+^ similarity – entropy area into eight rectangular subsets. For each subset, we calculated the ratio of patient count per disease stage over the total number of patients in the cohort in order to obtain the fraction for each disease stage per subset. We performed cohort randomization controls to assess the significance of the observed distributions. New disease stage annotations were randomly assigned 1,000 times to each of the 92 patients, maintaining the overall patient distribution and only changing disease stage annotation. Random sampling permutation was carried out using the MATLAB random sampling tool (The Mathworks Inc, Version 14, Statistics Toolbox). Disease stage fraction distribution was analysed for each of the 1,000 random patient cohorts as described above. Results are presented as histograms of frequency over fraction (%). Actual fractions observed in the patient cohort (GSE4170) are indicated (red lines) (Supplementary Fig. S11).

## Additional Information

**How to cite this article**: Brehme, M. *et al.* Combined Population Dynamics and Entropy Modelling Supports Patient Stratification in Chronic Myeloid Leukemia. *Sci. Rep.* **6**, 24057; doi: 10.1038/srep24057 (2016).

## Supplementary Material

Supplementary Information
