# Supplementary material for: Combined Population Dynamics and Entropy Modelling Supports Patient Stratification in Chronic Myeloid Leukemia
Source: Sci Rep. 2016 Apr 6;6:24057. doi: 10.1038/srep24057 (PMC4822142; doi:10.1038/srep24057)
Supplement: Supplementary Information [file srep24057-s1.pdf]

SUPPLEMENTARY INFORMATION

Combined Population Dynamics and Entropy Modelling Supports Patient Stratification in Chronic Myeloid Leukemia

Marc Brehme [1,6], Steffen Koschmieder [2,6], Maryam Montazeri [1], Mhairi Copland [3], Vivian G. Oehler [4], Jerald P. Radich [4], Tim H. Brümmendorf [2,6], and Andreas Schuppert [1,5,6,*]

[1] Joint Research Center for Computational Biomedicine (JRC-COMBINE), RWTH Aachen University, 52062 Aachen, Germany

[2] Department of Hematology, Oncology, Hemostaseology, and Stem Cell Transplantation, Faculty of Medicine, RWTH Aachen University, 52074 Aachen, Germany

[3] Paul O'Gorman Leukaemia Research Centre, Institute of Cancer Sciences, College of Medical Veterinary and Life Sciences, University of Glasgow, Glasgow G12 8QQ, United Kingdom

[4] Fred Hutchinson Cancer Research Center, Seattle, WA 98109, USA

[5] Aachen Institute for Advanced Study in Computational Engineering Science (AICES), RWTH Aachen University, 52062 Aachen, Germany

[6] MB and SK as well as THB and AS contributed equally to this work

[*] Corresponding author

INVENTORY OF SUPPLEMENTARY INFORMATION

Supplementary Figures and Figure Legends S1 through S11

Figure S1. Separation of population-based effects using stem and progenitor cell data from primary CML patients.

Figure S2. Separation of CML disease stages based on patient cell mixtures.

Figure S3. Significant correlation of patient-derived vs. simulated CD34$^+$ status.

Figure S4. Singularities of simulated gene expression entropies coincide with cell population mixing during hematopoietic evolution.

Figure S5. Significant correlation of patient-derived vs. simulated gene expression entropy.

Figure S6. CP patient disease stage and progression risk assessment.

Figure S7. CD34 ratio compartment cut-off and T1-T2 chronic phase boundary

Figure S8. Differential gene expression during CML disease progression.

Figure S9. Robust differential expression between CML disease stages.

Figure S10. Effect of cohort size on differential gene expression between CML disease stages

Figure S11. Patient cohort randomization controls.

Supplementary Tables S1 through S2

Table S1 GO Enrichment analysis of genes differentially up-regulated between early and late CP CML.

Table S2. GO Enrichment analysis of genes differentially down-regulated between early and late CP CML.

Supplementary Table Legends

Supplementary Resources and Methods

1. **Population dynamic model**
2. **Hematopoietic cell populations**
3. **CML patient cohort**
4. **Bootstrapping analyses**

Supplementary References

Supplementary Figures and Figure Legends

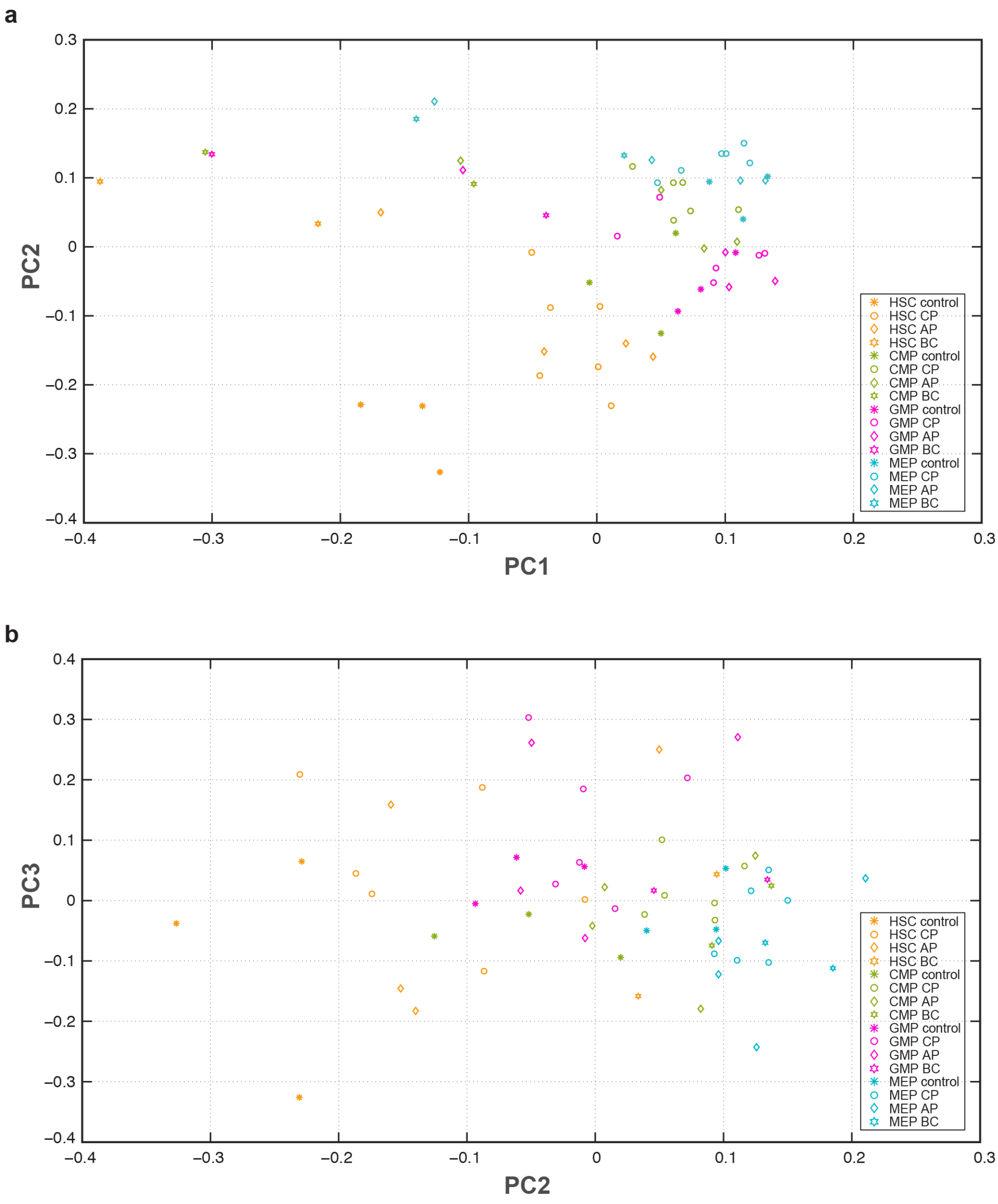

Figure S1. Separation of population-based effects using stem and progenitor cell data from primary CML patients. Principal Component Analysis (PCA) of genome-wide expression in 4 stem- and progenitor cell populations in CML stages and controls. Two-dimensional representation of principal components PC1 vs. PC2 (**a**) and PC2 vs. PC3 (**b**) are shown. Data as in Fig. 2. CP: chronic phase, AP: accelerated phase (blast count), $AP_{cyto:}$ accelerated phase (clonal evolution), BC: blast crisis, HSC: hematopoietic stem cell, MEP: megakaryocyte-erythroid progenitor, GMP: granulocyte-macrophage progenitor, CMP: common myeloid progenitor. Dataset GSE47927 [1].

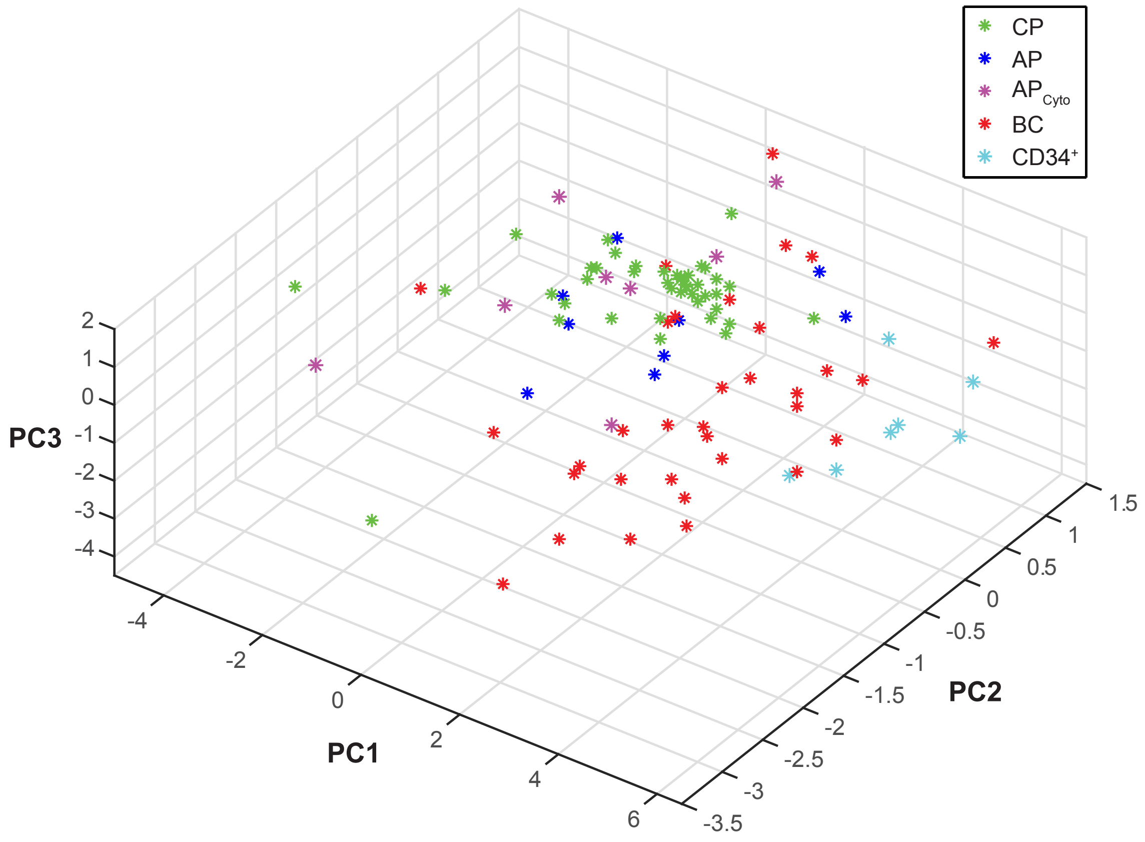

Figure S2. Separation of CML disease stages based on patient cell mixtures. Projection of the gene expression from clinical samples of 91 patients in different CML disease stages based on dataset GSE4170 [2] onto principal components (PCs) show differentiation between disease stages (CP and BC), in contrast to subpopulation-specific data. Each asterisk represents single patients with disease stage indicated by colour as follows: CP (green), AP_{cyto} (purple), AP (blue), and BC (red). 6 purified normal $CD34^+$ cell samples were included in the analysis as reference for similarity with immature blasts in BP (turquoise).

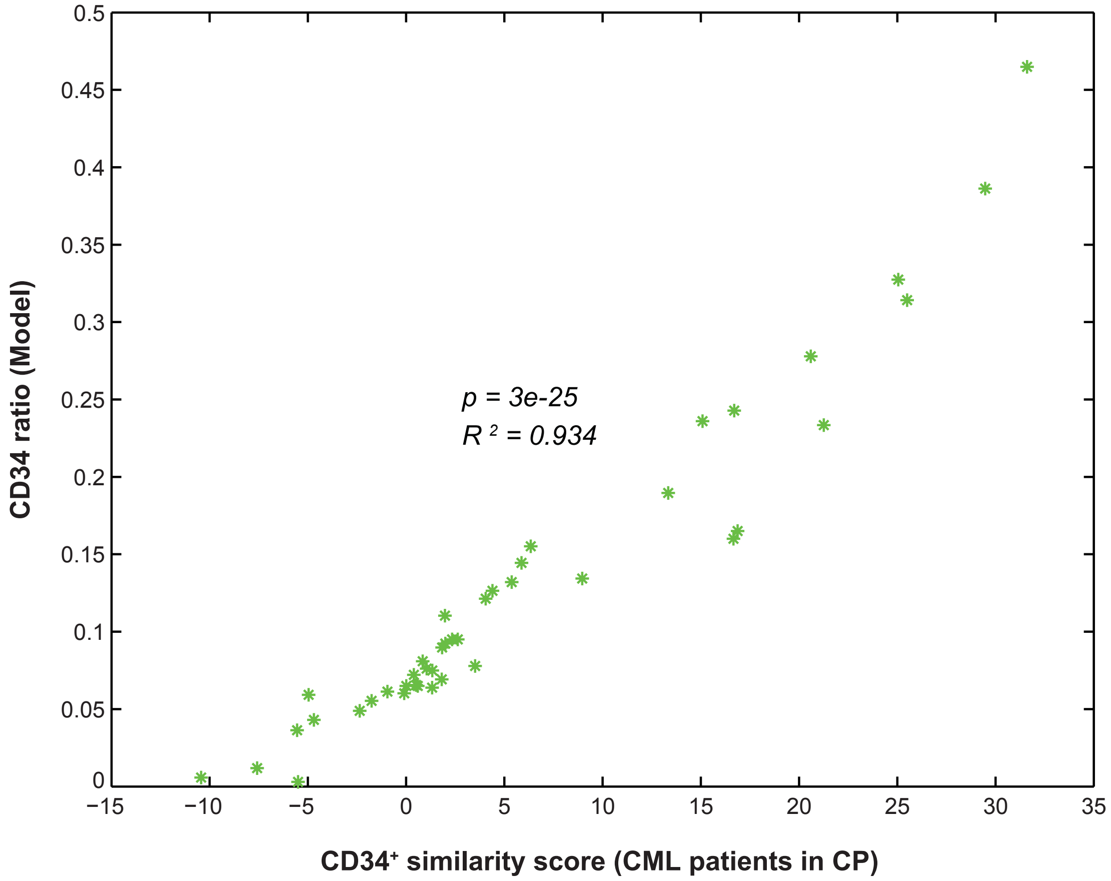

Figure S3. Significant correlation of patient-derived vs. simulated CD34+ status. Correlation of patient CD34+ similarity scores of CD34 expression in mixed patient samples compared to purified CD34+ cell populations (x-axis), with CD34 ratio inferred from the model (R^2 = 0.934, p = 3e-25).

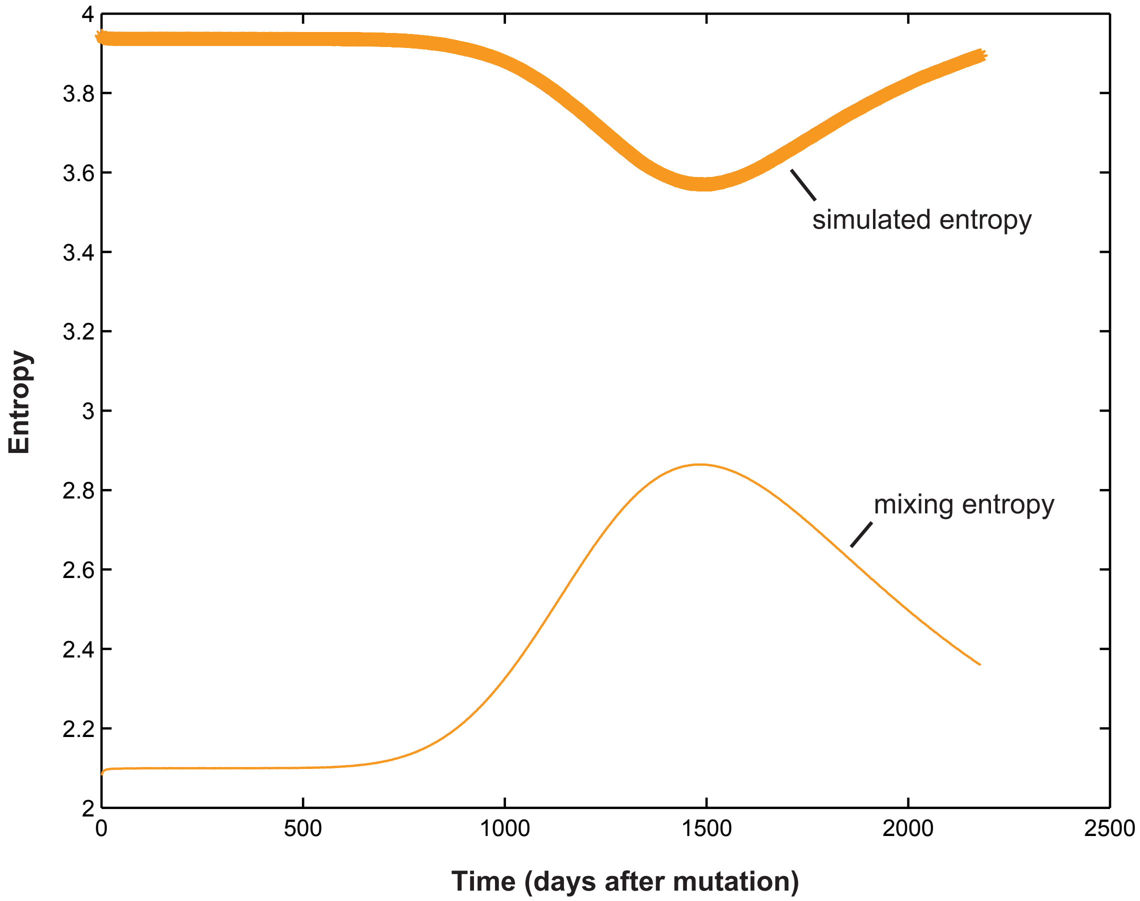

Figure S4. Singularities of simulated gene expression entropies coincide with cell population mixing during hematopoietic evolution. Simulated entropy of randomized gene expression (considering the stable subset of low-IR genes, upper curve) displays a non-monotonic dynamic trend with a singular minimum point. Equally, simulation of entropy of cell population mixing throughout hematopoietic evolution (mixing entropy, lower curve) shows a singular maximum point that coincides with the minimum of gene expression entropy derived from the model.

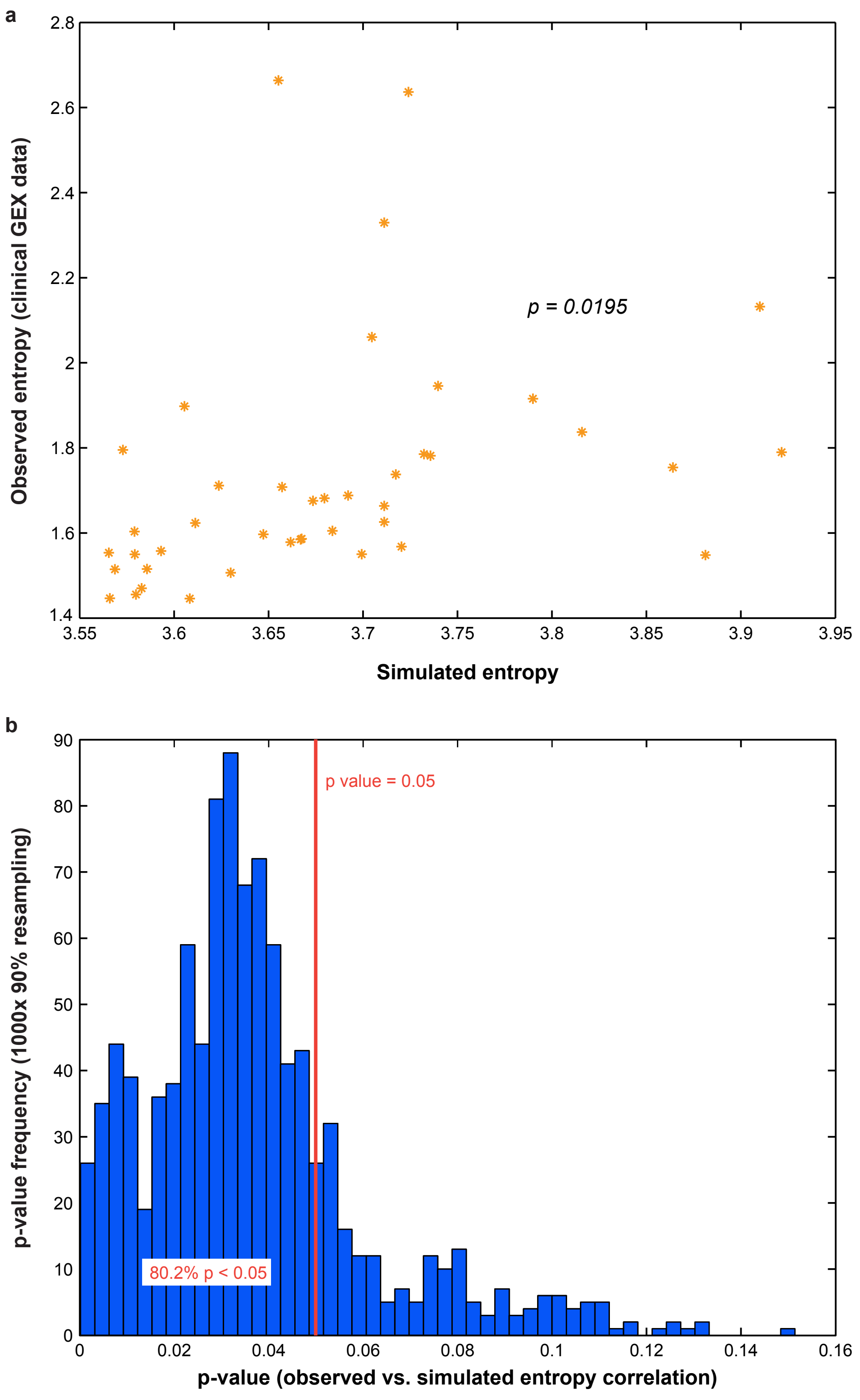

Figure S5. Significant correlation of patient-derived vs. simulated gene expression entropy. a. Patient gene expression entropy is significantly correlated with simulated entropy ($p = 0.0195$). **b.** Bootstrapping analysis, randomly dropping 10% of the data points in 1000 iterations without replacement. P value frequency distribution for corresponding correlations between simulated and observed entropies considering remaining 90% of data points is shown.

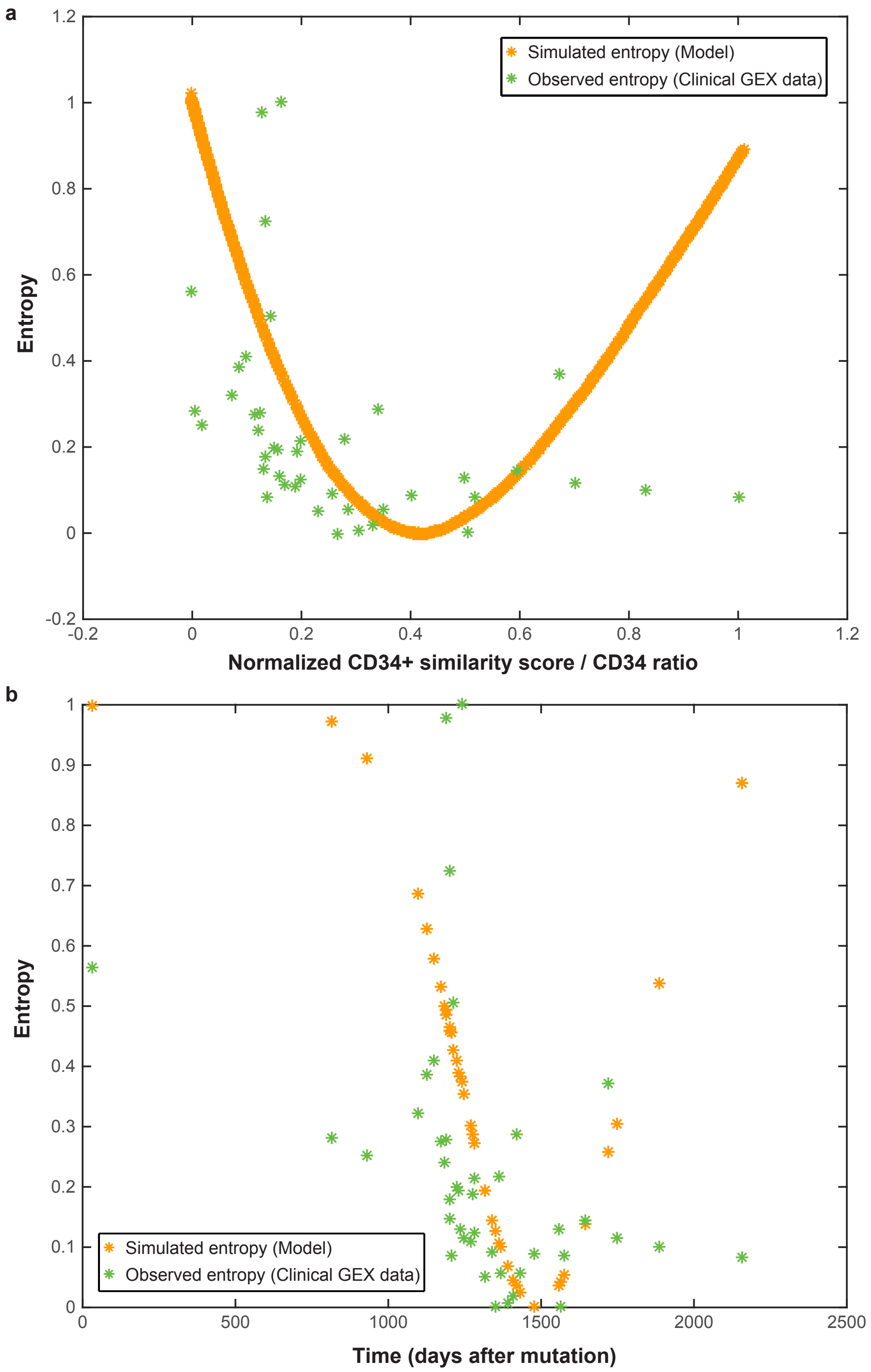

Figure S6. CP patient disease stage and progression risk assessment. a. Data points represent 42 CP patients (Fig. 4A). Simulated and observed entropies were normalized to interval [0 1], matching $CD34^+$ similarity score normalization. **b.** Entropies were normalized to interval [0 1] to highlight similarity of observed and simulated entropy minima with respect to disease time.

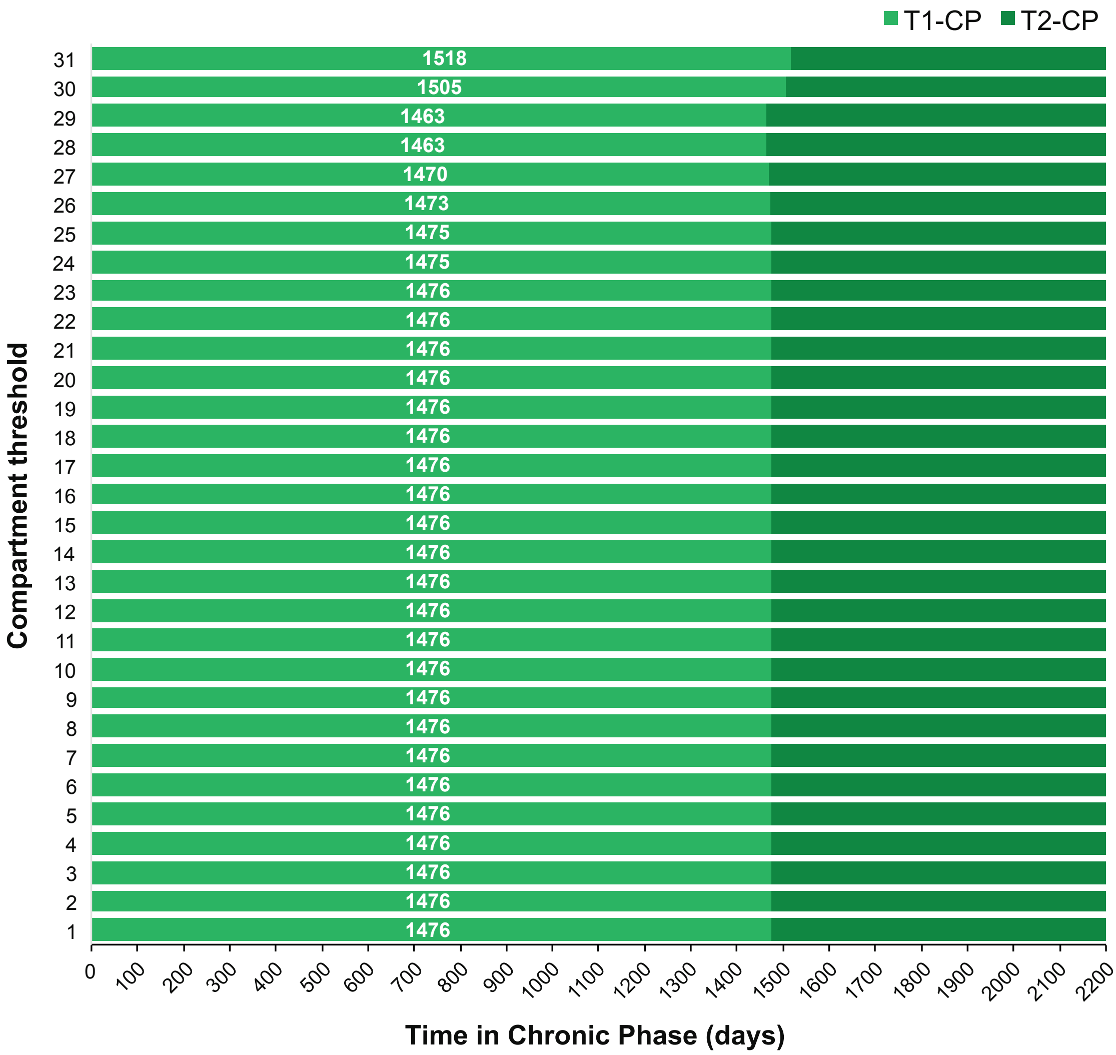

Figure S7. CD34 ratio compartment cut-off and T1-T2 chronic phase boundary. CD34 ratios are calculated considering the full range of possible cut-offs across the 32 hematopoietic compartments [3]. The impact on the boundary separating T1-CP from T2-CP around 4 years is shown, indicating overall robustness of the model.

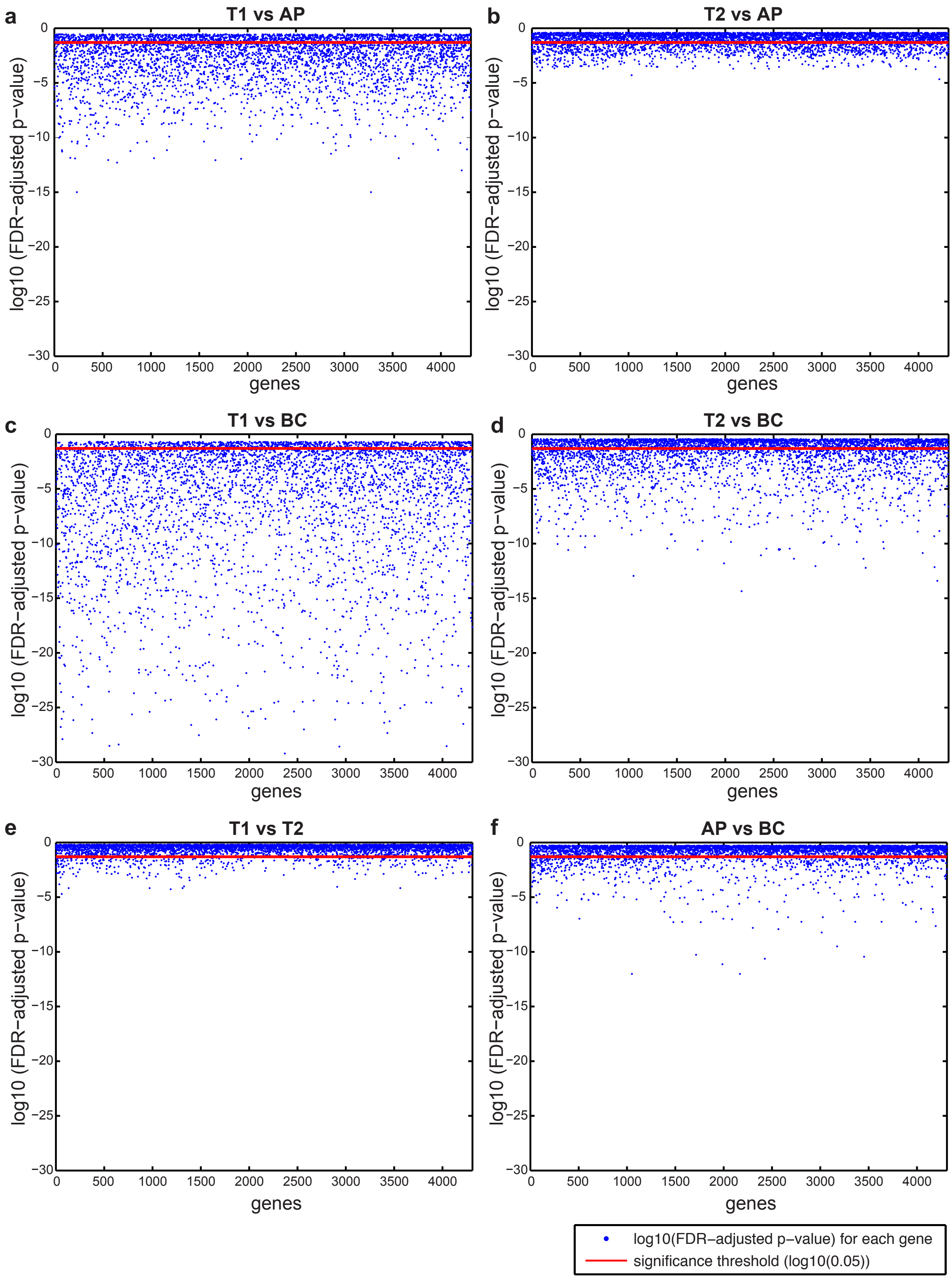

Figure S8. Differential gene expression during CML disease progression.

a. - f. Significance of differential expression of each gene (blue dots) between CML disease stages is plotted as log10 p-value. A cut-off of $p < 0.05$ (FDR-adjusted p-value) is indicated (red line). T1 = “early”, T2 = “late” chronic phase (CP), AP = advanced phase, BC = blast crisis.

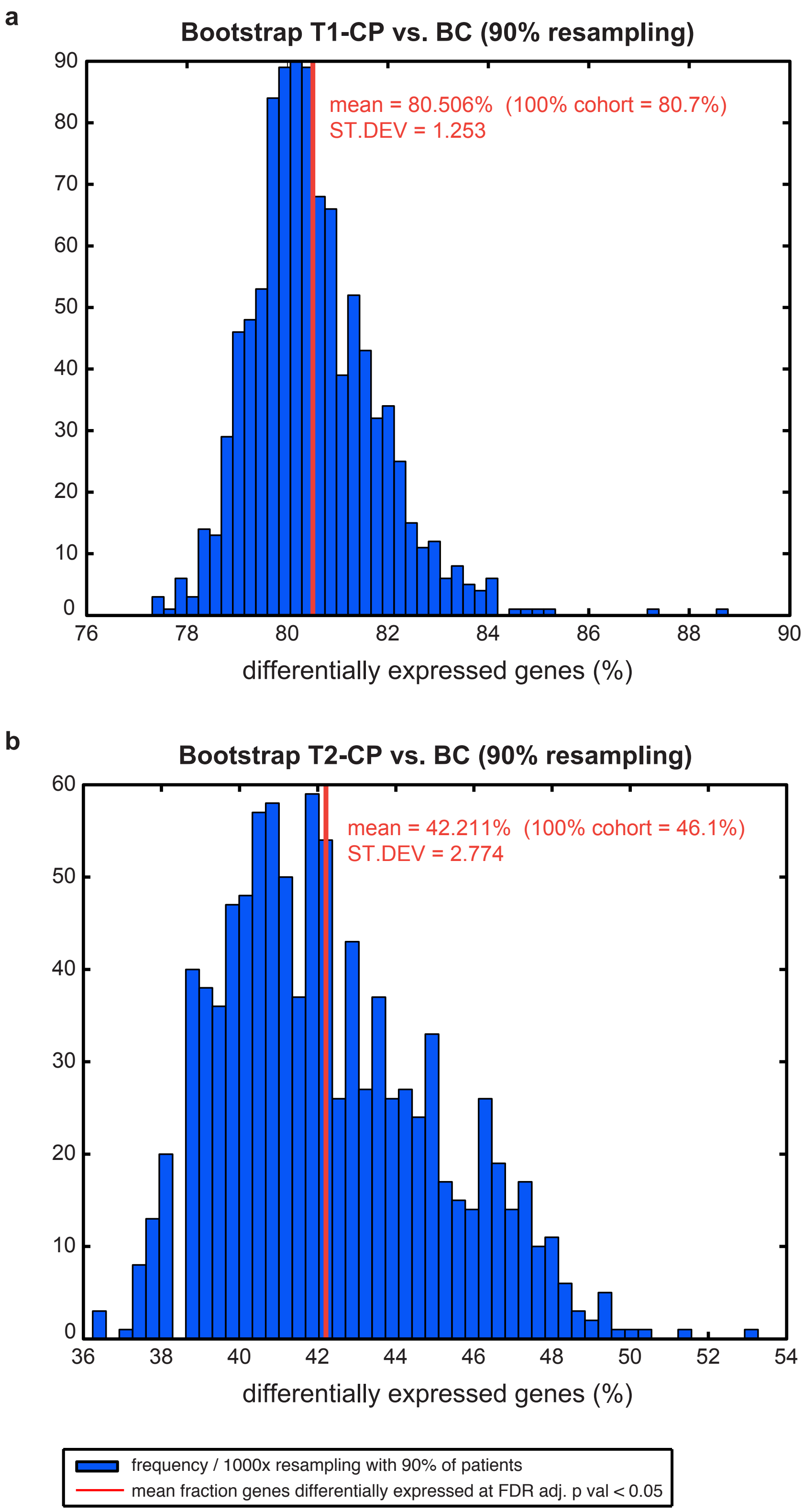

Figure S9. Robust differential expression between CML disease stages. T1 ("early") and "T2 ("late") CP patients are significantly different in terms of gene expression (FDR-adjusted $p < 0.05$, two-sided *t*-test). Frequency of fractions of differentially expressed genes at 1000 iterations of random sub-sampling considering 90% of patients are shown for **a.** T1-CP vs. BC, and **b.** T2-CP vs. BC.

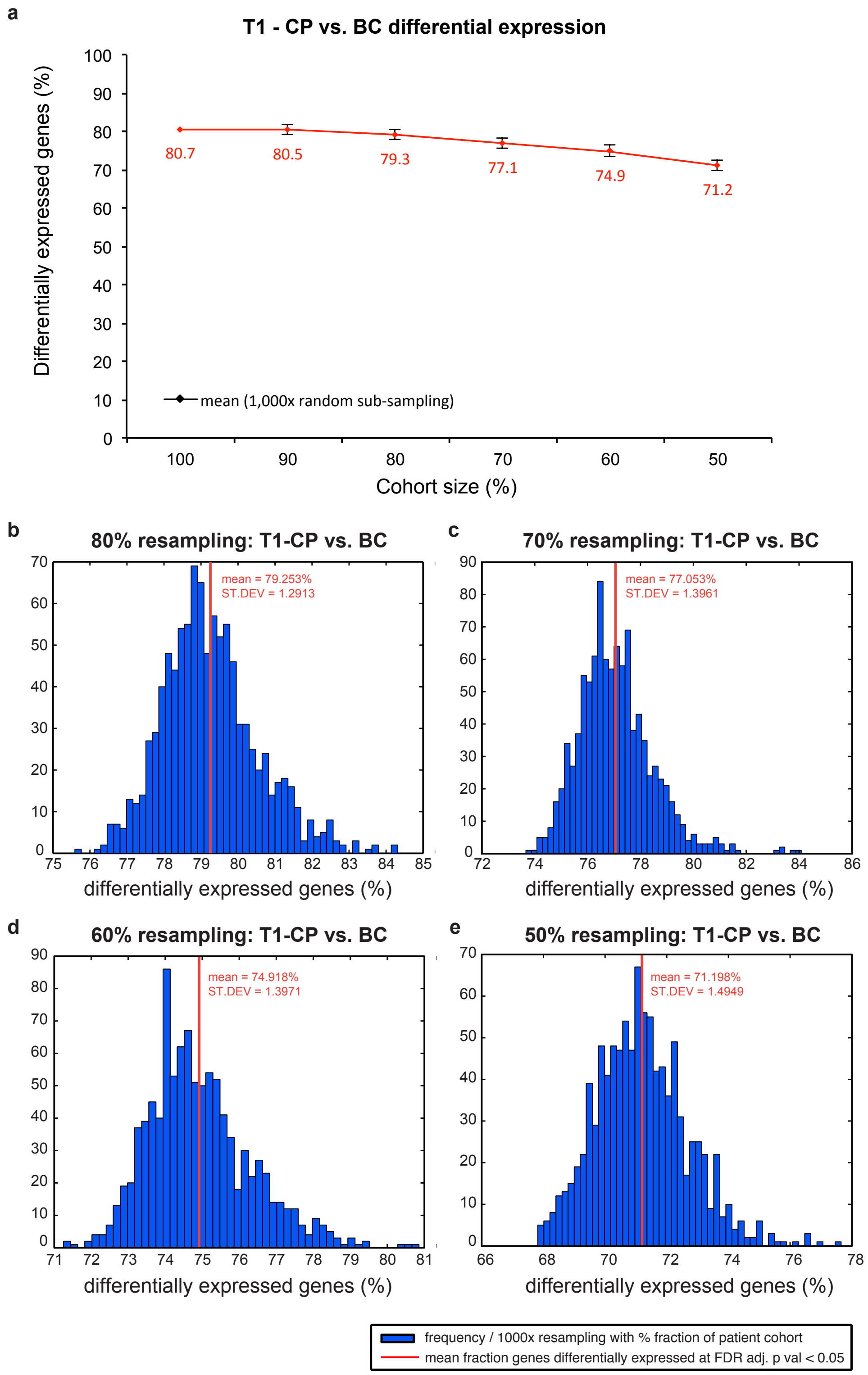

Figure S10. Effect of cohort size on differential gene expression between CML disease stages. a. Mean fractions of genes differentially expressed between T1-CP and BC at 1000 iterations of sub-sampling with 80%, 70%, 60%, and 50% of patients **b - e.** Frequency of fractions of genes differentially expressed between T1-CP and BC after 1000 iterations of sub-sampling considering 80% (**b**), 70% (**c**), 60% (**d**), and 50% (**e**) of patients.

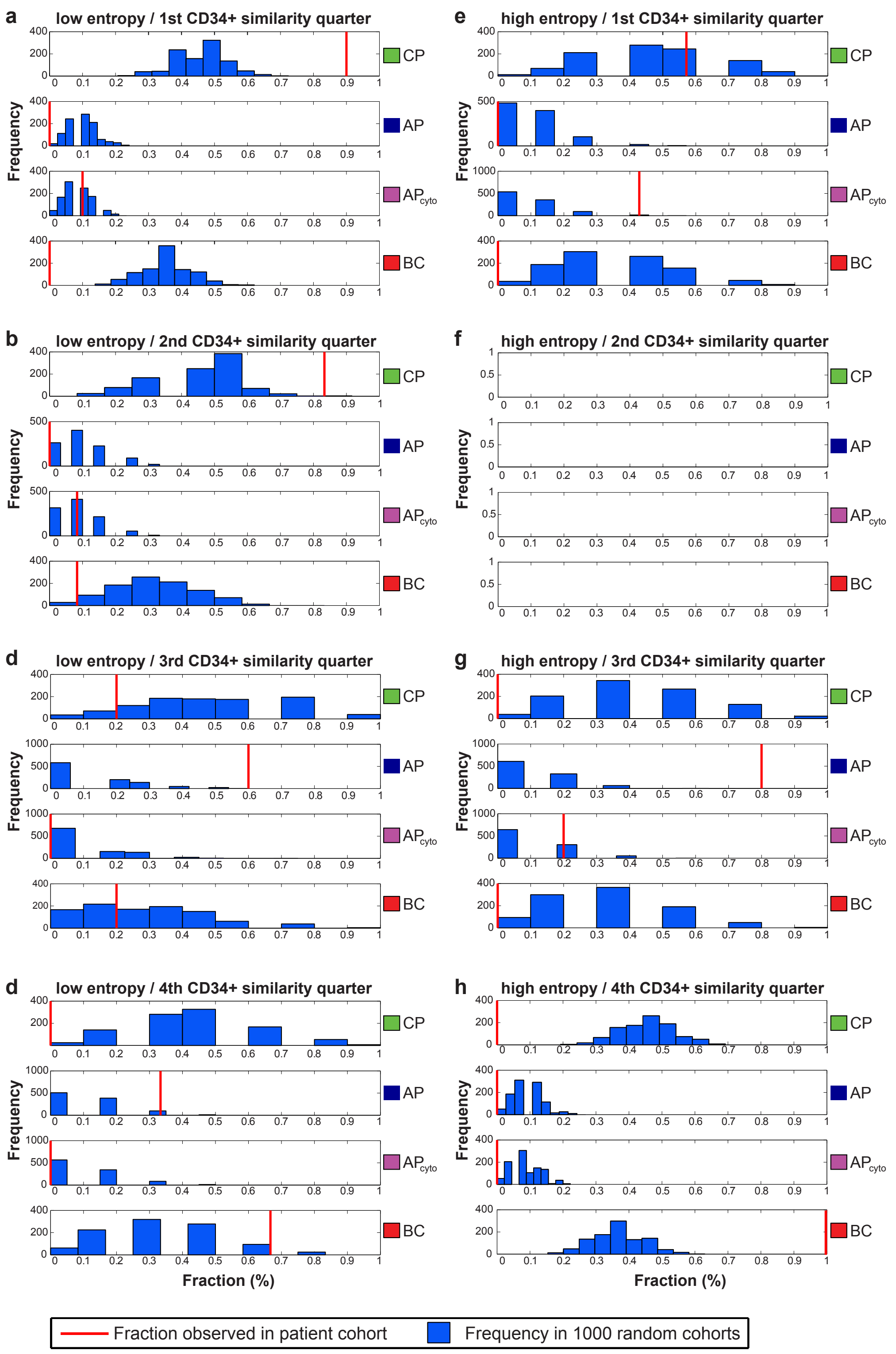

Figure S11. Patient cohort randomization control. Disease stage fractions (%)for 42 CP, 9 AP, 8 AP_{cyto}, and 28 BC CML patients, divided into $CD34^+$ similarity score quarters and low vs. high entropy, compared to 1,000 analogous patient cohorts with randomly assigned disease stages. **a - h**. Frequency distribution of disease stage fraction (%) for each of the eight sub-spaces. Fractions per disease stage observed in the patient cohort (GSE4170) indicated as red lines [2].

Supplementary Tables

Table S1

Analysis Type:	PANTHER Overrepresentation Test (release 20150430)
Annotation Version and Release Date:	GO Ontology database Released 2015-08-06
Analyzed List:	Upregulated between T1 and T2 (Homo sapiens)
Reference List:	bioDBnet_db2db_150807035413_758562527.txt (Homo sapiens)
Bonferroni correction:	TRUE

GO biological process complete	bioDBnet_db2db_150807035413_758562527.txt - REFLIST (4111)	UP (203)	UP (expected)	UP (over/under)	UP (fold Enrichment)	UP (P-value)	DYNAMICS
RNA catabolic process	66	19	3.26	+	> 5	5.62E-06	UP
viral gene expression	36	14	1.78	+	> 5	2.37E-05	UP
protein localization to endoplasmic reticulum	38	14	1.88	+	> 5	4.63E-05	UP
multi-organism metabolic process	38	14	1.88	+	> 5	4.63E-05	UP
viral transcription	34	13	1.68	+	> 5	1.03E-04	UP
nuclear-transcribed mRNA catabolic process	55	16	2.72	+	> 5	1.05E-04	UP
mRNA catabolic process	56	16	2.77	+	> 5	1.34E-04	UP
protein targeting to membrane	50	15	2.47	+	> 5	1.98E-04	UP
nuclear-transcribed mRNA catabolic process, nonsense-mediated decay	36	13	1.78	+	> 5	1.99E-04	UP
positive regulation of cellular protein catabolic process	67	17	3.31	+	> 5	2.65E-04	UP
SRP-dependent cotranslational protein targeting to membrane	31	12	1.53	+	> 5	3.20E-04	UP
cotranslational protein targeting to membrane	31	12	1.53	+	> 5	3.20E-04	UP
protein targeting to ER	31	12	1.53	+	> 5	3.20E-04	UP
establishment of protein localization to endoplasmic reticulum	31	12	1.53	+	> 5	3.20E-04	UP
positive regulation of proteolysis involved in cellular protein catabolic process	63	16	3.11	+	> 5	6.54E-04	UP
ribosome biogenesis	41	12	2.02	+	> 5	5.97E-03	UP
cellular protein complex disassembly	50	13	2.47	+	> 5	7.83E-03	UP
translational elongation	51	13	2.52	+	> 5	9.70E-03	UP
protein stabilization	37	11	1.83	+	> 5	1.44E-02	UP
nuclear export	32	10	1.58	+	> 5	2.66E-02	UP
nucleobase-containing compound transport	48	12	2.37	+	> 5	2.93E-02	UP
signal transduction involved in mitotic G1 DNA damage checkpoint	40	11	1.98	+	> 5	2.99E-02	UP
signal transduction involved in mitotic cell cycle checkpoint	40	11	1.98	+	> 5	2.99E-02	UP
DNA damage response, signal transduction by p53 class mediator resulting in cell cycl	40	11	1.98	+	> 5	2.99E-02	UP
signal transduction involved in mitotic DNA integrity checkpoint	40	11	1.98	+	> 5	2.99E-02	UP
signal transduction involved in mitotic DNA damage checkpoint	40	11	1.98	+	> 5	2.99E-02	UP
intracellular signal transduction involved in G1 DNA damage checkpoint	40	11	1.98	+	> 5	2.99E-02	UP
signal transduction involved in DNA damage checkpoint	41	11	2.02	+	> 5	3.76E-02	UP
signal transduction involved in DNA integrity checkpoint	41	11	2.02	+	> 5	3.76E-02	UP
signal transduction involved in cell cycle checkpoint	42	11	2.07	+	> 5	4.70E-02	UP
positive regulation of cell cycle arrest	49	12	2.42	+	4.96	3.60E-02	UP
translational initiation	60	14	2.96	+	4.73	1.08E-02	UP
anaphase	60	14	2.96	+	4.73	1.08E-02	UP
mitotic anaphase	60	14	2.96	+	4.73	1.08E-02	UP
regulation of protein stability	62	14	3.06	+	4.57	1.57E-02	UP
antigen processing and presentation of exogenous peptide antigen	73	16	3.6	+	4.44	4.49E-03	UP
antigen processing and presentation of peptide antigen	79	17	3.9	+	4.36	2.61E-03	UP
antigen processing and presentation of exogenous antigen	79	17	3.9	+	4.36	2.61E-03	UP
viral life cycle	75	16	3.7	+	4.32	6.36E-03	UP
positive regulation of protein catabolic process	90	19	4.44	+	4.28	7.29E-04	UP
signal transduction by p53 class mediator	67	14	3.31	+	4.23	3.74E-02	UP
nucleobase-containing compound catabolic process	109	22	5.38	+	4.09	1.57E-04	UP
mitotic cell cycle checkpoint	75	15	3.7	+	4.05	2.96E-02	UP
mitotic M phase	76	15	3.75	+	4	3.46E-02	UP
translation	102	20	5.04	+	3.97	1.08E-03	UP
M phase	77	15	3.8	+	3.95	4.03E-02	UP
heterocycle catabolic process	125	24	6.17	+	3.89	9.11E-05	UP
regulation of cellular protein catabolic process	111	21	5.48	+	3.83	9.50E-04	UP
regulation of proteolysis involved in cellular protein catabolic process	106	20	5.23	+	3.82	1.97E-03	UP
antigen processing and presentation	97	18	4.79	+	3.76	9.67E-03	UP
regulation of protein catabolic process	147	27	7.26	+	3.72	2.74E-05	UP
establishment of protein localization to organelle	121	22	5.97	+	3.68	9.36E-04	UP
nucleocytoplasmic transport	88	16	4.35	+	3.68	4.69E-02	UP
cytoplasmic transport	223	40	11.01	+	3.63	6.67E-09	UP
cellular nitrogen compound catabolic process	123	22	6.07	+	3.62	1.23E-03	UP
aromatic compound catabolic process	130	23	6.42	+	3.58	7.81E-04	UP
organic cyclic compound catabolic process	140	24	6.91	+	3.47	7.29E-04	UP
regulation of protein ubiquitination	106	18	5.23	+	3.44	3.24E-02	UP
mitotic cell cycle phase transition	130	22	6.42	+	3.43	3.09E-03	UP
peptide metabolic process	148	25	7.31	+	3.42	5.18E-04	UP
regulation of protein modification by small protein conjugation or removal	113	19	5.58	+	3.41	2.05E-02	UP
RNA processing	138	23	6.81	+	3.38	2.19E-03	UP
protein targeting	139	23	6.86	+	3.35	2.48E-03	UP
cell cycle phase transition	133	22	6.57	+	3.35	4.48E-03	UP
amide biosynthetic process	127	21	6.27	+	3.35	8.09E-03	UP
protein localization to organelle	170	28	8.39	+	3.34	1.40E-04	UP
viral process	262	43	12.94	+	3.32	1.51E-08	UP
multi-organism cellular process	264	43	13.04	+	3.3	1.93E-08	UP
microtubule-based process	124	20	6.12	+	3.27	2.09E-02	UP
nuclear division	120	19	5.93	+	3.21	4.75E-02	UP
positive regulation of proteolysis	153	24	7.56	+	3.18	3.53E-03	UP
cellular component disassembly	173	27	8.54	+	3.16	7.40E-04	UP
interspecies interaction between organisms	283	44	13.97	+	3.15	4.74E-08	UP
symbiosis, encompassing mutualism through parasitism	283	44	13.97	+	3.15	4.74E-08	UP
positive regulation of cell cycle	135	21	6.67	+	3.15	2.07E-02	UP
mitotic cell cycle process	239	36	11.8	+	3.05	1.17E-05	UP
negative regulation of transferase activity	140	21	6.91	+	3.04	3.59E-02	UP
positive regulation of protein transport	152	22	7.51	+	2.93	3.75E-02	UP
mitotic cell cycle	265	37	13.09	+	2.83	5.02E-05	UP
generation of precursor metabolites and energy	165	23	8.15	+	2.82	4.20E-02	UP
intracellular protein transport	245	34	12.1	+	2.81	2.53E-04	UP
regulation of cellular protein localization	176	24	8.69	+	2.76	3.80E-02	UP
negative regulation of cellular component organization	206	28	10.17	+	2.75	6.59E-03	UP
cytokine-mediated signaling pathway	194	26	9.58	+	2.71	2.09E-02	UP
cellular amide metabolic process	199	26	9.83	+	2.65	3.28E-02	UP
regulation of protein transport	271	35	13.38	+	2.62	8.79E-04	UP
cell cycle process	352	45	17.38	+	2.59	1.40E-05	UP
cellular response to cytokine stimulus	263	33	12.99	+	2.54	3.94E-03	UP
regulation of establishment of protein localization	301	37	14.86	+	2.49	1.25E-03	UP
response to cytokine	310	38	15.31	+	2.48	8.94E-04	UP
negative regulation of cell death	361	44	17.83	+	2.47	8.99E-05	UP
regulation of protein localization	333	40	16.44	+	2.43	6.77E-04	UP
organonitrogen compound biosynthetic process	325	39	16.05	+	2.43	1.03E-03	UP
positive regulation of cellular component organization	395	46	19.5	+	2.36	1.55E-04	UP
apoptotic process	441	51	21.78	+	2.34	2.76E-05	UP
macromolecule localization	592	68	29.23	+	2.33	3.16E-08	UP
negative regulation of cellular protein metabolic process	356	41	17.58	+	2.33	1.40E-03	UP
negative regulation of protein metabolic process	384	43	18.96	+	2.27	1.44E-03	UP
vesicle-mediated transport	370	41	18.27	+	2.24	3.79E-03	UP
organic substance transport	553	61	27.31	+	2.23	3.15E-06	UP
positive regulation of transport	348	38	17.18	+	2.21	1.48E-02	UP
regulation of organelle organization	352	38	17.38	+	2.19	1.93E-02	UP
protein complex subunit organization	456	48	22.52	+	2.13	1.53E-03	UP
regulation of apoptotic process	563	58	27.8	+	2.09	1.20E-04	UP
negative regulation of macromolecule metabolic process	745	76	36.79	+	2.07	3.44E-07	UP
regulation of programmed cell death	569	58	28.1	+	2.06	1.75E-04	UP
regulation of cell death	594	59	29.33	+	2.01	3.21E-04	UP
negative regulation of gene expression	436	43	21.53	+	2	3.91E-02	UP
gene expression	975	96	48.15	+	1.99	1.05E-09	UP
regulation of cellular localization	447	44	22.07	+	1.99	3.13E-02	UP
small molecule metabolic process	851	83	42.02	+	1.98	2.61E-07	UP
organonitrogen compound metabolic process	606	59	29.92	+	1.97	6.48E-04	UP
negative regulation of metabolic process	846	82	41.78	+	1.96	5.14E-07	UP
RNA metabolic process	805	78	39.75	+	1.96	2.06E-06	UP
cellular response to organic substance	821	79	40.54	+	1.95	2.06E-06	UP

**negative regulation of cellular metabolic process**	**757**	**73**	**37.38**	**+**	**1.95**	**1.32E-05**	**UP**
**cellular component biogenesis**	**574**	**55**	**28.34**	**+**	**1.94**	**3.42E-03**	**UP**
**regulation of transport**	**663**	**63**	**32.74**	**+**	**1.92**	**4.89E-04**	**UP**
**positive regulation of cellular protein metabolic process**	**562**	**53**	**27.75**	**+**	**1.91**	**9.52E-03**	**UP**
**single-organism transport**	**974**	**90**	**48.1**	**+**	**1.87**	**4.30E-07**	**UP**
**positive regulation of protein metabolic process**	**596**	**55**	**29.43**	**+**	**1.87**	**1.11E-02**	**UP**
**nucleic acid metabolic process**	**949**	**87**	**46.86**	**+**	**1.86**	**1.77E-06**	**UP**
**cellular component assembly**	**546**	**50**	**26.96**	**+**	**1.85**	**4.66E-02**	**UP**
**macromolecular complex subunit organization**	**628**	**57**	**31.01**	**+**	**1.84**	**1.14E-02**	**UP**
**multi-organism process**	**781**	**70**	**38.57**	**+**	**1.82**	**6.83E-04**	**UP**
**cellular response to chemical stimulus**	**999**	**89**	**49.33**	**+**	**1.8**	**4.33E-06**	**UP**
**response to endogenous stimulus**	**709**	**63**	**35.01**	**+**	**1.8**	**5.42E-03**	**UP**
**regulation of cellular protein metabolic process**	**861**	**75**	**42.52**	**+**	**1.76**	**6.10E-04**	**UP**
**cellular nitrogen compound biosynthetic process**	**919**	**79**	**45.38**	**+**	**1.74**	**3.95E-04**	**UP**
**nucleobase-containing compound biosynthetic process**	**808**	**69**	**39.9**	**+**	**1.73**	**5.61E-03**	**UP**
**cellular macromolecule biosynthetic process**	**952**	**81**	**47.01**	**+**	**1.72**	**3.79E-04**	**UP**
**macromolecule biosynthetic process**	**985**	**83**	**48.64**	**+**	**1.71**	**3.59E-04**	**UP**
**heterocycle metabolic process**	**1238**	**104**	**61.13**	**+**	**1.7**	**1.34E-06**	**UP**
**regulation of protein metabolic process**	**929**	**78**	**45.87**	**+**	**1.7**	**1.40E-03**	**UP**
**heterocycle biosynthetic process**	**835**	**70**	**41.23**	**+**	**1.7**	**8.95E-03**	**UP**
**organelle organization**	**812**	**68**	**40.1**	**+**	**1.7**	**1.43E-02**	**UP**
**cellular aromatic compound metabolic process**	**1248**	**104**	**61.63**	**+**	**1.69**	**2.21E-06**	**UP**
**organic cyclic compound biosynthetic process**	**890**	**74**	**43.95**	**+**	**1.68**	**5.15E-03**	**UP**
**regulation of localization**	**858**	**71**	**42.37**	**+**	**1.68**	**1.17E-02**	**UP**
**cell surface receptor signaling pathway**	**835**	**69**	**41.23**	**+**	**1.67**	**1.87E-02**	**UP**
**regulation of catalytic activity**	**812**	**67**	**40.1**	**+**	**1.67**	**2.95E-02**	**UP**
**organic cyclic compound metabolic process**	**1344**	**110**	**66.37**	**+**	**1.66**	**1.14E-06**	**UP**
**aromatic compound biosynthetic process**	**840**	**69**	**41.48**	**+**	**1.66**	**2.31E-02**	**UP**
**regulation of signal transduction**	**891**	**72**	**44**	**+**	**1.64**	**2.31E-02**	**UP**
**cellular biosynthetic process**	**1285**	**103**	**63.45**	**+**	**1.62**	**3.03E-05**	**UP**
**response to chemical**	**1476**	**116**	**72.88**	**+**	**1.59**	**2.92E-06**	**UP**
**organic substance biosynthetic process**	**1319**	**103**	**65.13**	**+**	**1.58**	**1.37E-04**	**UP**
**regulation of molecular function**	**984**	**77**	**48.59**	**+**	**1.58**	**3.01E-02**	**UP**
**biosynthetic process**	**1344**	**104**	**66.37**	**+**	**1.57**	**1.81E-04**	**UP**
**regulation of macromolecule biosynthetic process**	**1066**	**82**	**52.64**	**+**	**1.56**	**2.37E-02**	**UP**
**regulation of signaling**	**1010**	**78**	**49.87**	**+**	**1.56**	**4.17E-02**	**UP**
**regulation of nitrogen compound metabolic process**	**1179**	**89**	**58.22**	**+**	**1.53**	**1.47E-02**	**UP**
**regulation of biosynthetic process**	**1155**	**87**	**57.03**	**+**	**1.53**	**2.32E-02**	**UP**
**signal transduction**	**1559**	**109**	**76.98**	**+**	**1.42**	**1.78E-02**	**UP**
**regulation of macromolecule metabolic process**	**1641**	**114**	**81.03**	**+**	**1.41**	**1.05E-02**	**UP**
**regulation of cellular metabolic process**	**1721**	**118**	**84.98**	**+**	**1.39**	**1.09E-02**	**UP**
**single organism signaling**	**1705**	**117**	**84.19**	**+**	**1.39**	**1.25E-02**	**UP**
**signaling**	**1705**	**117**	**84.19**	**+**	**1.39**	**1.25E-02**	**UP**
**cell communication**	**1729**	**118**	**85.38**	**+**	**1.38**	**1.43E-02**	**UP**
**regulation of primary metabolic process**	**1675**	**114**	**82.71**	**+**	**1.38**	**3.29E-02**	**UP**
**cellular response to stimulus**	**2027**	**137**	**100.09**	**+**	**1.37**	**6.08E-04**	**UP**
**regulation of metabolic process**	**1907**	**129**	**94.17**	**+**	**1.37**	**3.18E-03**	**UP**
**regulation of cellular process**	**2777**	**167**	**137.13**	**+**	**1.22**	**8.95E-03**	**UP**
mRNA metabolic process	139	31	6.86	+	4.52	1.50E-08	UP / DOWN
establishment of protein localization to membrane	83	17	4.1	+	4.15	5.08E-03	UP / DOWN
peptide biosynthetic process	108	21	5.33	+	3.94	6.08E-04	UP / DOWN
cellular macromolecule catabolic process	234	43	11.55	+	3.72	3.60E-10	UP / DOWN
macromolecule catabolic process	292	46	14.42	+	3.19	9.11E-09	UP / DOWN
protein localization to membrane	129	20	6.37	+	3.14	3.72E-02	UP / DOWN
cellular protein catabolic process	157	23	7.75	+	2.97	1.89E-02	UP / DOWN
single-organism intracellular transport	364	53	17.97	+	2.95	2.55E-09	UP / DOWN
proteolysis involved in cellular protein catabolic process	153	22	7.56	+	2.91	4.15E-02	UP / DOWN
single-organism membrane organization	245	33	12.1	+	2.73	8.11E-04	UP / DOWN
organic substance catabolic process	512	68	25.28	+	2.69	3.16E-11	UP / DOWN
membrane organization	295	39	14.57	+	2.68	8.17E-05	UP / DOWN
intracellular transport	424	55	20.94	+	2.63	7.69E-08	UP / DOWN
regulation of catabolic process	280	36	13.83	+	2.6	6.35E-04	UP / DOWN
cellular catabolic process	483	61	23.85	+	2.56	1.21E-08	UP / DOWN
establishment of localization in cell	571	72	28.2	+	2.55	4.99E-11	UP / DOWN
negative regulation of apoptotic process	336	42	16.59	+	2.53	1.01E-04	UP / DOWN
negative regulation of programmed cell death	337	42	16.64	+	2.52	1.10E-04	UP / DOWN
cellular macromolecule localization	371	46	18.32	+	2.51	2.31E-05	UP / DOWN
cellular protein localization	371	46	18.32	+	2.51	2.31E-05	UP / DOWN
protein transport	355	44	17.53	+	2.51	5.49E-05	UP / DOWN
single-organism cellular localization	282	35	13.93	+	2.51	2.23E-03	UP / DOWN
establishment of protein localization	388	47	19.16	+	2.45	3.12E-05	UP / DOWN
cell cycle	442	53	21.83	+	2.43	3.51E-06	UP / DOWN
catabolic process	595	71	29.38	+	2.42	1.32E-09	UP / DOWN
cellular localization	677	80	33.43	+	2.39	2.71E-11	UP / DOWN
negative regulation of catalytic activity	314	37	15.51	+	2.39	3.47E-03	UP / DOWN
negative regulation of molecular function	403	46	19.9	+	2.31	2.82E-04	UP / DOWN
protein localization	511	58	25.23	+	2.3	3.37E-06	UP / DOWN
programmed cell death	449	51	22.17	+	2.3	5.01E-05	UP / DOWN
cell death	466	51	23.01	+	2.22	1.68E-04	UP / DOWN
death	470	51	23.21	+	2.2	2.21E-04	UP / DOWN
regulation of response to stress	549	53	27.11	+	1.96	4.67E-03	UP / DOWN
transport	1133	103	55.95	+	1.84	1.19E-08	UP / DOWN
single-organism localization	1036	94	51.16	+	1.84	3.17E-07	UP / DOWN
establishment of localization	1174	106	57.97	+	1.83	6.18E-09	UP / DOWN
response to organic substance	1097	98	54.17	+	1.81	2.09E-07	UP / DOWN
regulation of cellular component organization	734	65	36.24	+	1.79	3.78E-03	UP / DOWN
cellular response to stress	639	56	31.55	+	1.77	4.21E-02	UP / DOWN
nucleobase-containing compound metabolic process	1165	101	57.53	+	1.76	4.87E-07	UP / DOWN
cellular nitrogen compound metabolic process	1407	120	69.48	+	1.73	1.94E-09	UP / DOWN
negative regulation of cellular process	1365	115	67.4	+	1.71	3.04E-08	UP / DOWN
localization	1431	120	70.66	+	1.7	7.15E-09	UP / DOWN
negative regulation of biological process	1487	123	73.43	+	1.68	6.85E-09	UP / DOWN
cellular protein metabolic process	1097	91	54.17	+	1.68	1.03E-04	UP / DOWN
nitrogen compound metabolic process	1532	125	75.65	+	1.65	9.70E-09	UP / DOWN
protein metabolic process	1309	101	64.64	+	1.56	4.28E-04	UP / DOWN
single-organism metabolic process	1511	114	74.61	+	1.53	7.39E-05	UP / DOWN
cellular macromolecule metabolic process	1825	136	90.12	+	1.51	3.50E-07	UP / DOWN
positive regulation of cellular process	1584	118	78.22	+	1.51	6.21E-05	UP / DOWN
regulation of response to stimulus	1236	92	61.03	+	1.51	1.61E-02	UP / DOWN
cellular component organization	1603	119	79.16	+	1.5	6.10E-05	UP / DOWN
cellular component organization or biogenesis	1616	119	79.8	+	1.49	1.04E-04	UP / DOWN
response to stress	1394	101	68.84	+	1.47	1.16E-02	UP / DOWN
system development	1505	106	74.32	+	1.43	2.03E-02	UP / DOWN
macromolecule metabolic process	2045	142	100.98	+	1.41	2.03E-05	UP / DOWN
multicellular organismal development	1647	113	81.33	+	1.39	2.50E-02	UP / DOWN
cellular metabolic process	2424	164	119.7	+	1.37	1.38E-07	UP / DOWN
positive regulation of biological process	1785	121	88.14	+	1.37	1.26E-02	UP / DOWN
single-organism developmental process	1803	120	89.03	+	1.35	4.35E-02	UP / DOWN
primary metabolic process	2454	162	121.18	+	1.34	4.02E-06	UP / DOWN
organic substance metabolic process	2559	163	126.36	+	1.29	1.06E-04	UP / DOWN
metabolic process	2744	168	135.5	+	1.24	1.20E-03	UP / DOWN
regulation of biological process	2909	171	143.65	+	1.19	2.90E-02	UP / DOWN
Unclassified	81	4	4	+	1	0.00E+00	UP / DOWN

**PROCESS**	**UP (%)**	**UP/DOWN (%)**	**DOWN (%)**
**IMMUNE/DEFENSE**	0.0	0.0	**30.8**
**DIFFERENTIATION/DEVELOPMENT**	0.0	0.0	**15.4**
DNA DAMAGE/CELL CYCLE/CHECKPOINT	**12.9**	1.5	0.0
**PROTEIN TRANSLATION/MODIFICATION/TRAFFICKING**	14.7	9.2	11.5
**METABOLIC**	14.7	20.0	7.7
BIOSYNTHETIC	8.6	1.5	0.0
**CATABOLIC**	9.2	12.3	11.5
SUM	10.8	11.3	9.6

Table S2

Analysis Type: PANTHER Overrepresentation Test (release 20150430)
Annotation Version and Release Date: GO Ontology database Released 2015-08-06
Analyzed List: Downregulated between T1 and T2 (Homo sapiens)
Reference List: bioDBnet_db2db_150807035413_758562527.txt (Homo sapiens)
Bonferroni correction: TRUE

GO biological process complete	bioDBnet_db2db_150807035413_758562527.txt - REFLIST (4111)	DOWN (199)	DOWN (expected)	DOWN (over/under)	DOWN (fold Enrichment)	DOWN (P-value)	DYNAMICS
**tRNA metabolic process**	**27**	**9**	**1.31**	**+**	**> 5**	**4.05E-02**	**DOWN**
**activation of innate immune response**	**116**	**19**	**5.62**	**+**	**3.38**	**2.22E-02**	**DOWN**
**innate immune response-activating signal transduction**	**116**	**19**	**5.62**	**+**	**3.38**	**2.22E-02**	**DOWN**
**protein ubiquitination**	**137**	**22**	**6.63**	**+**	**3.32**	**5.19E-03**	**DOWN**
**protein catabolic process**	**175**	**27**	**8.47**	**+**	**3.19**	**6.15E-04**	**DOWN**
**positive regulation of innate immune response**	**138**	**21**	**6.68**	**+**	**3.14**	**2.11E-02**	**DOWN**
**ubiquitin-dependent protein catabolic process**	**134**	**20**	**6.49**	**+**	**3.08**	**4.79E-02**	**DOWN**
**regulation of innate immune response**	**171**	**25**	**8.28**	**+**	**3.02**	**4.97E-03**	**DOWN**
**positive regulation of defense response**	**175**	**25**	**8.47**	**+**	**2.95**	**7.50E-03**	**DOWN**
**protein modification by small protein conjugation**	**166**	**23**	**8.04**	**+**	**2.86**	**3.33E-02**	**DOWN**
**protein modification by small protein conjugation or removal**	**193**	**25**	**9.34**	**+**	**2.68**	**4.08E-02**	**DOWN**
**regulation of cellular catabolic process**	**246**	**30**	**11.91**	**+**	**2.52**	**1.55E-02**	**DOWN**
**small GTPase mediated signal transduction**	**263**	**32**	**12.73**	**+**	**2.51**	**7.30E-03**	**DOWN**
**regulation of defense response**	**305**	**36**	**14.76**	**+**	**2.44**	**2.96E-03**	**DOWN**
**regulation of immune response**	**400**	**40**	**19.36**	**+**	**2.07**	**3.86E-02**	**DOWN**
**regulation of immune system process**	**577**	**56**	**27.93**	**+**	**2**	**8.35E-04**	**DOWN**
**movement of cell or subcellular component**	**581**	**55**	**28.12**	**+**	**1.96**	**2.53E-03**	**DOWN**
**locomotion**	**545**	**51**	**26.38**	**+**	**1.93**	**1.07E-02**	**DOWN**
**generation of neurons**	**606**	**55**	**29.33**	**+**	**1.87**	**9.54E-03**	**DOWN**
**neurogenesis**	**641**	**56**	**31.03**	**+**	**1.8**	**2.42E-02**	**DOWN**
**cell differentiation**	**1185**	**89**	**57.36**	**+**	**1.55**	**6.75E-03**	**DOWN**
**cellular developmental process**	**1207**	**90**	**58.43**	**+**	**1.54**	**7.72E-03**	**DOWN**
**positive regulation of metabolic process**	**1253**	**92**	**60.65**	**+**	**1.52**	**1.07E-02**	**DOWN**
**anatomical structure development**	**1680**	**121**	**81.32**	**+**	**1.49**	**5.58E-05**	**DOWN**
**developmental process**	**1834**	**127**	**88.78**	**+**	**1.43**	**1.90E-04**	**DOWN**
**single-organism cellular process**	**3319**	**183**	**160.66**	**+**	**1.14**	**4.13E-02**	**DOWN**
establishment of protein localization to membrane	83	19	4.02	+	4.73	1.53E-04	DOWN / UP
peptide biosynthetic process	108	18	5.23	+	3.44	3.16E-02	DOWN / UP
cellular protein catabolic process	157	25	7.6	+	3.29	1.05E-03	DOWN / UP
mRNA metabolic process	139	22	6.73	+	3.27	6.56E-03	DOWN / UP
proteolysis involved in cellular protein catabolic process	153	24	7.41	+	3.24	2.45E-03	DOWN / UP
protein localization to membrane	129	20	6.24	+	3.2	2.76E-02	DOWN / UP
cellular macromolecule catabolic process	234	36	11.33	+	3.18	3.84E-06	DOWN / UP
macromolecule catabolic process	292	41	14.13	+	2.9	3.08E-06	DOWN / UP
single-organism cellular localization	282	35	13.65	+	2.56	1.37E-03	DOWN / UP
single-organism membrane organization	245	29	11.86	+	2.45	4.06E-02	DOWN / UP
membrane organization	295	34	14.28	+	2.38	1.08E-02	DOWN / UP
regulation of catabolic process	280	32	13.55	+	2.36	2.68E-02	DOWN / UP
cellular catabolic process	483	55	23.38	+	2.35	4.45E-06	DOWN / UP
establishment of protein localization	388	42	18.78	+	2.24	2.85E-03	DOWN / UP
negative regulation of catalytic activity	314	34	15.2	+	2.24	4.13E-02	DOWN / UP
organic substance catabolic process	512	55	24.78	+	2.22	3.58E-05	DOWN / UP
negative regulation of apoptotic process	336	36	16.26	+	2.21	2.70E-02	DOWN / UP
negative regulation of programmed cell death	337	36	16.31	+	2.21	2.88E-02	DOWN / UP
single-organism intracellular transport	364	38	17.62	+	2.16	2.57E-02	DOWN / UP
protein transport	355	37	17.18	+	2.15	3.64E-02	DOWN / UP
catabolic process	595	61	28.8	+	2.12	2.31E-05	DOWN / UP
cellular macromolecule localization	371	38	17.96	+	2.12	3.98E-02	DOWN / UP
cellular protein localization	371	38	17.96	+	2.12	3.98E-02	DOWN / UP
cell cycle	442	45	21.4	+	2.1	5.54E-03	DOWN / UP
negative regulation of molecular function	403	41	19.51	+	2.1	1.90E-02	DOWN / UP
intracellular transport	424	42	20.52	+	2.05	2.74E-02	DOWN / UP
establishment of localization in cell	571	55	27.64	+	1.99	1.44E-03	DOWN / UP
cell death	466	45	22.56	+	1.99	2.26E-02	DOWN / UP
protein localization	511	49	24.74	+	1.98	9.11E-03	DOWN / UP
death	470	45	22.75	+	1.98	2.83E-02	DOWN / UP
programmed cell death	449	43	21.73	+	1.98	4.79E-02	DOWN / UP
regulation of response to stress	549	52	26.58	+	1.96	5.71E-03	DOWN / UP
cellular localization	677	62	32.77	+	1.89	1.12E-03	DOWN / UP
Unclassified	81	7	3.92	+	1.79	0.00E+00	DOWN / UP
cellular response to stress	639	55	30.93	+	1.78	4.75E-02	DOWN / UP
regulation of cellular component organization	734	61	35.53	+	1.72	3.86E-02	DOWN / UP
establishment of localization	1174	93	56.83	+	1.64	2.07E-04	DOWN / UP
transport	1133	88	54.84	+	1.6	1.75E-03	DOWN / UP
single-organism localization	1036	79	50.15	+	1.58	2.41E-02	DOWN / UP
cellular component organization or biogenesis	1616	123	78.23	+	1.57	6.01E-07	DOWN / UP
cellular component organization	1603	121	77.6	+	1.56	2.06E-06	DOWN / UP
negative regulation of biological process	1487	112	71.98	+	1.56	3.03E-05	DOWN / UP
negative regulation of cellular process	1365	103	66.08	+	1.56	2.57E-04	DOWN / UP
cellular protein metabolic process	1097	83	53.1	+	1.56	1.56E-02	DOWN / UP
response to organic substance	1097	82	53.1	+	1.54	3.11E-02	DOWN / UP
cellular nitrogen compound metabolic process	1407	104	68.11	+	1.53	6.51E-04	DOWN / UP
nucleobase-containing compound metabolic process	1165	85	56.39	+	1.51	4.96E-02	DOWN / UP
nitrogen compound metabolic process	1532	111	74.16	+	1.5	4.28E-04	DOWN / UP
positive regulation of cellular process	1584	114	76.68	+	1.49	3.24E-04	DOWN / UP
localization	1431	103	69.27	+	1.49	3.42E-03	DOWN / UP
regulation of response to stimulus	1236	89	59.83	+	1.49	4.39E-02	DOWN / UP
protein metabolic process	1309	94	63.36	+	1.48	2.09E-02	DOWN / UP
positive regulation of biological process	1785	127	86.41	+	1.47	2.73E-05	DOWN / UP
response to stress	1394	99	67.48	+	1.47	1.45E-02	DOWN / UP
single-organism metabolic process	1511	107	73.14	+	1.46	3.73E-03	DOWN / UP
multicellular organismal development	1647	115	79.73	+	1.44	1.65E-03	DOWN / UP
system development	1505	105	72.85	+	1.44	1.21E-02	DOWN / UP
single-organism developmental process	1803	125	87.28	+	1.43	2.84E-04	DOWN / UP
cellular macromolecule metabolic process	1825	124	88.34	+	1.4	1.38E-03	DOWN / UP
cellular metabolic process	2424	160	117.34	+	1.36	4.89E-07	DOWN / UP
primary metabolic process	2454	159	118.79	+	1.34	4.85E-06	DOWN / UP
macromolecule metabolic process	2045	131	98.99	+	1.32	1.64E-02	DOWN / UP
organic substance metabolic process	2559	160	123.87	+	1.29	1.19E-04	DOWN / UP
metabolic process	2744	166	132.83	+	1.25	4.78E-04	DOWN / UP
regulation of biological process	2909	168	140.82	+	1.19	2.64E-02	DOWN / UP

PROCESS	UP (%)	UP/DOWN (%)	DOWN (%)
**IMMUNE/DEFENSE**	0.0	0.0	**30.8**
**DIFFERENTIATION/DEVELOPMENT**	0.0	0.0	**15.4**
DNA DAMAGE/CELL CYCLE/CHECKPOINT	**12.9**	1.5	0.0
**PROTEIN TRANSLATION/MODIFICATION/TRAFFICKING**	14.7	9.2	11.5
**METABOLIC**	14.7	20.0	7.7
BIOSYNTHETIC	8.6	1.5	0.0
**CATABOLIC**	9.2	12.3	11.5
SUM	10.8	11.3	9.6

Supplementary Table Legends

Table S1. GO Enrichment analysis of genes differentially up-regulated between early and late CP CML. Table shows GO terms (biological process) significantly enriched ($p < 0.05$, Bonferroni correction) amongst genes found differentially up-regulated from T1 (early) to T2 (late) CP of CML, ordered by fold enrichment in decreasing order. GO terms exclusively enriched amongst up-regulated genes are highlighted in bold on top and separated by a red line from GO terms that were also found enriched amongst genes that are down-regulated between T1- and T2 CP.

Table S2. GO Enrichment analysis of genes differentially down-regulated between early and late CP CML. Table shows GO terms (biological process) significantly enriched ($p < 0.05$, Bonferroni correction) amongst genes found differentially down-regulated from T1 (early) to T2 (late) CP of CML, ordered by fold enrichment in decreasing order. GO terms exclusively enriched amongst down-regulated genes are highlighted in bold on top and separated by a red line from GO terms that were also found enriched amongst genes that are up-regulated between T1- and T2 CP.

Supplementary Resources and Methods

1. Population dynamic model

Disease progression during CML, i.e. clonal expansion of CML cells, is modelled according to a mathematical model by Dingli et al [4]. Healthy haematopoiesis is a process following a hierarchical multi-compartmental structure connecting hematopoietic stem cells (HSCs) to terminally differentiated cells, where cells either differentiate or self-renew. An active pool of 400 HSCs is responsible for normal marrow output with a differentiation property of $\varepsilon_0 \approx 0.85$, which is equal across compartments, while self-renewal probability is 1 - ε. Both probabilities are considered constant across healthy haematopoiesis at a replication rate of $r \approx 1.26$ over approximately 31 divisions *K* between the HSC and the circulating compartments. For further details on the multi-compartment model of normal haematopoiesis referred to in this study see the original publication by Dingli et al [3]. The number of active HSCs is not increased in CML and is estimated to remain constant at $N_i \approx 400$, while patients do have increased counts of myeloid progenitors. CML is diagnosed when bone marrow output exceeds 10^{12} cells per day as a consequence of reduced ε. According to the model, average clonal dynamics are approximated so that the number of cells in each compartment *i* ≥ 1 changes according the differential equation $\dot{N}_i = -d_i \times N_i + b_{i-1} \times N_{i-1}$, where $d_i = (2\varepsilon - 1) \times r_i$ represents the rate at which cells are leaving compartment *i*, and $b_{i-1} = 2 \times \varepsilon \times r_{i-1}$ indicates the rate at which cells originating in compartment *i* -1 enter compartment *i* . In the event of a cancer mutation in the HSC compartment, healthy HSCs are reduced to $N_0 - 1$ so that for normal cells $\dot{N}_i = -d_i \times N_i + b_{i-1} \times N_{i-1}$ at $\varepsilon_0 \approx 0.85$, whereas for CML cells (N_i^{CML}) we consider $\varepsilon_{CML} < \varepsilon_0$, indicating a lower probability of differentiation in CML. Starting with one single CML HSC, disease expansion, assessed at hands of the growth of the BCR-ABL/BCR ratio, takes almost 6 years until it becomes clinically evident at $\varepsilon_{CML} = 0.72$ (>10^{12} cells bone marrow output).

2. Hematopoietic cell sub-populations

Gene expression and principal component analyses (PCA) related to Figure 2, Supplementary Figure 1 online, and Supplementary Figure 2 online were carried out using data from $CD34^+$ enriched and flow-sorted CML stem and progenitor cell population samples derived from 12 CML patients and healthy controls as deposited

in the NCBI Gene Expression Omnibus (GEO) database under accession number GSE47927, provided by Copland M, and Irvine DA in 2013 [1]. Cell populations considered in the analyses include hematopoietic stem cells (HSCs), common myeloid progenitors (CMPs), granulocyte-macrophage progenitors (GMPs), and megakaryocyte-erythroid progenitor cells (MEPs) obtained from six patients in chronic phase (CP), four patients in accelerated phase (AP), and from two blast crisis patients (BC), compared to matching populations from three healthy volunteers. Following antibody staining cells were sorted into sub-populations on a FACS Aria cell sorter as follows: [HSC] $CD34^+$, $CD38^-$, lin^-, $CD45RA^-$, $CD90^+$; [MPP] $CD34^+$, $CD38^-$, lin^-, $CD45RA^-$, $CD90^-$; [CMP] $CD34^+$, $CD38^+$, lin^-, $CD45RA^-$, $CD123^+$; [GMP] $CD34^+$, $CD38^+$, lin^-, $CD45RA^+$, $CD123^+$, and [MEP] $CD34^+$, $CD38^+$, lin^-, $CD45RA^-$, $CD123^-$. For further details on sample preparation and experimental procedures we refer to the original publications as cited above.

3. CML patient cohort

CML patient data analysed in this study related to Figures 3 through 6 are based on a study by Radich and co-workers [2]. For the identification of patient disease progression features we analysed the gene expression dataset associated with this publication as deposited in the GEO database under the accession GSE4170. This dataset includes genome-wide expression data from 42 CML patients in chronic phase (CP), 9 patients in advanced phase (AP), 8 patients in advanced phase based on additional clonal cytogenetic changes but without increased blast count (AP_{cyto}), as well as 28 patients in blast crisis (BC), measured on 60-mer oligonucleotide Rosetta Inpharmatics / Merck Pharmaceuticals Human 25k v2.2.1 microarrays. Samples from the different CML stages were hybridized against the pool of chronic phases of samples. Patient samples originated from the Fred Hutchinson Cancer Research Center, the Southwest Oncology Group (SWOG) Myeloid Repository, the University of Oregon Health Sciences Center, the University of California, Los Angeles, or the University of Chicago. RNA extraction was either performed immediately, or in the case of liquid nitrogen preserved samples, after thawing. CP, AP, and BC stages were assigned based on the criteria defined by Sokal et al. and the International Bone Marrow Transplant Registry, where CP < 10% blasts, AP = 10 - 30% blasts or < 10% blasts with clonal evolution (additional cytogenetic changes), and BC > 30% blasts [5,6]. Patients in CP with no available blast count were assigned

blast count = 1. The dataset includes normal $CD34^+$ enriched cells as control samples, which we considered in our analysis (Supplementary Fig. S2 online).

4. Bootstrapping analyses

Supplementary Figure S5:

Significance of the p value for the correlation between simulated and clinically observed entropy of gene expression was challenged through 1,000 iterations of random sub-sampling, removing 10% of data points in each iteration without replacement, and calculating the p value of the resulting correlation on the remaining 90% of data points. Resulting p value frequencies for 1,000 iterations are indicated.

Supplementary Figures S9 and S10:

The fractions (%) of genes differentially expressed between CML disease stages (see Supplementary Figure S8) were identified at a cut-off of $p < 0.05$ (FDR-adjusted p value) and significance of differential expression was assessed by 2-sided t-test as described in the methods section accompanying this manuscript (MATLAB statistical toolbox). To assess robustness of the fractions of differentially expressed genes identified between the CML disease stages using this approach, random sub-sampling considering 90% of the patient cohort was performed at 1,000 iterations. Frequencies of fractions of differentially expressed genes identified across all iterations at $p < 0.05$ (FDR-adjusted p value) are shown and the mean and associated standard deviation (ST.DEV) were determined. The same methodology was applied to simulate effect of changes in patient cohort size on the mean fraction of genes differentially expressed between CML chronic phase (T1-CP) and blast crisis (BC). To this end, random sub-sampling at 1,000 iterations across patient cohorts reduced in size by discrete increments of 10% were performed, each time considering the remaining 90% of patients to determine the fractions (%) of genes differentially expressed genes. Corresponding mean fractions of differentially expressed genes and associated ST.DEV were identified accordingly.

Supplementary References

1. Cramer-Morales, K. *et al.* Personalized synthetic lethality induced by targeting RAD52 in leukemias identified by gene mutation and expression profile. *Blood* **122,** 1293–1304 (2013).
2. Radich, J. P. *et al.* Gene expression changes associated with progression and response in chronic myeloid leukemia. *Proc. Natl. Acad. Sci. U. S. A.* **103,** 2794–2799 (2006).
3. Dingli, D., Traulsen, A. & Pacheco, J. M. Compartmental architecture and dynamics of hematopoiesis. *PloS One* **2,** e345 (2007).
4. Dingli, D., Traulsen, A. & Pacheco, J. M. Chronic Myeloid Leukemia: Origin, Development, Response to Therapy, and Relapse. *Clin. Leuk.* **2,** 133–139 (2008).
5. Sokal, J. E. *et al.* Prognostic discrimination in 'good-risk' chronic granulocytic leukemia. *Blood* **63,** 789–799 (1984).
6. Savage, D. G., Szydlo, R. M., Chase, A., Apperley, J. F. & Goldman, J. M. Bone marrow transplantation for chronic myeloid leukaemia: the effects of differing criteria for defining chronic phase on probabilities of survival and relapse. *Br. J. Haematol.* **99,** 30–35 (1997).
